# Parkin regulates IGF2BP3 through ubiquitination in the tumourigenesis of cervical cancer

**DOI:** 10.1002/ctm2.1457

**Published:** 2023-10-25

**Authors:** Xin Sun, Guiqin Ye, Jiuzhou Li, Huafeng Shou, Gongxun Bai, Jianbin Zhang

**Affiliations:** ^1^ Department of Medical Oncology Cancer Center Key Laboratory of Tumor Molecular Diagnosis and Individualized Medicine of Zhejiang Province Zhejiang Provincial People's Hospital (Affiliated People's Hospital, Hangzhou Medical College) Hangzhou China; ^2^ Basic Medical Sciences Hangzhou Medical College Hangzhou China; ^3^ Department of Neurosurgery Binzhou People's Hospital Binzhou China; ^4^ Department of Gynecology Zhejiang Provincial People's Hospital (Affiliated People's Hospital, Hangzhou Medical College) Binzhou China; ^5^ Key Laboratory of Rare Earth Optoelectronic Materials and Devices of Zhejiang Province, College of Optical and Electronic Technology China Jiliang University Hangzhou China

**Keywords:** cervical cancer, IGF2BP3, mitophagy, parkin, ubiquitination

## Abstract

**Background:**

Insulin‐like growth Factor 2 mRNA‐binding protein 3 (IGF2BP3) is a highly conserved RNA‐binding protein and plays a critical role in regulating posttranscriptional modifications.

**Methods:**

Immunoprecipitation was used to examine the interaction of Parkin and IGF2BP3. Mass spectrometry was performed to identify the ubiquitination sites of IGF2BP3. RNA‐immunoprecipitation was conducted to examine the target genes of IGF2BP3. Xenograft mouse model was constructed to determine the tumorigenesis of IGF2BP3.

**Results:**

IGF2BP3 expression is negatively correlated with Parkin expression in human cervical cancer cells and tissues. Parkin directly interacts with IGF2BP3, and overexpression of Parkin causes the proteasomal degradation of IGF2BP3, while knockdown of PARK2 increases the protein levels of IGF2BP3. Mechanistically, in vivo and in vitro ubiquitination assays demonstrated that Parkin is able to ubiquitinate IGF2BP3. Moreover, the ubiquitination site of IGF2BP3 was identified at K213 in the first KH domain of IGF2BP3. IGF2BP3 mutation results in the loss of its oncogenic function as an m6A reader, resulting in the inactivation of the phosphoinositide 3‐kinase (PI3K) and mitogen‐activated protein kinase (MAPK) signalling pathways. In addition, IGF2BP3 mutation results in the attenuation of Parkin‐mediated mitophagy, indicating its inverse role in regulating Parkin. Consequently, the tumourigenesis of cervical cancer is also inhibited by IGF2BP3 mutation.

**Conclusion:**

IGF2BP3 is ubiquitinated and regulated by the E3 ubiquitin ligase Parkin in human cervical cancer and ubiquitination modification plays an important role in modulating IGF2BP3 function. Thus, understanding the role of IGF2BP3 in tumourigenesis could provide new insights into cervical cancer therapy.

## Graphical abstract

- IGF2BP3 is ubiquitinated and regulated by the E3 ubiquitin ligase Parkin in human cervical cancer.
- Parkin directly interacts with IGF2BP3, and overexpression of Parkin causes the proteasomal degradation of IGF2BP3.
- IGF2BP3 mutation results in the loss of its oncogenic function as an m^6^A reader.
- IGF2BP3 mutation results in the attenuation of Parkin‐mediated mitophagy.

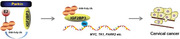

## 1 INTRODUCTION

Cervical cancer is the most challenging malignant tumour to treat among women worldwide.^1^ Human papillomavirus (HPV) infection is a diagnostic indicator of cervical cancer, and women with HPV infection have a high risk of cervical cancer.^2^ However, the occurrence of cervical cancer cannot be fully elucidated by HPV infection. Most recently, RNA‐binding proteins (RBPs) have been reported to be involved in the occurrence of cervical cancer.^3^ At the post‐transcriptional level, RBPs recognise and bind to special RNAs and regulate target RNA processing. RBPs play a critical role in regulating post‐transcriptional modifications. To date, 1542 RBPs have been identified and are approximately 7.5% of the proteome.^4^ IGF2BP has three isoforms, and these isoforms exhibit sequence homology. Among these isoforms, IGF2BP1 and IGF2BP3 share 73% sequence similarity.^5^ As zipcode‐binding proteins, they are composed of two RNA‐recognition motifs in their N‐terminus and 4 K‐homology RNA‐binding domains in their C‐terminus. Functionally, RRMs mainly stabilise IGF2BP–RNA complexes,^6^ while the KH domains are involved in RNA binding and ribonucleoprotein (RNP) granule formation.^7^ IGF2BPs mainly localise in the cytoplasm, where they form RNP granules and accumulate near the nucleus.^8^

IGF2BPs are highly expressed in the embryogenesis stage, and IGF2BP1 and IGF2BP3, but not IGF2BP2, become absent with human development.^9^ At the post‐transcriptional level, IGF2BPs regulate their target RNAs in various aspects,^5^ such as splicing, stability and subcellular localisation. IGF2BPs have their own RNA‐recognition motifs that mediate their binding to target RNAs at the 5′ untranslated region (5′‐UTR), 3′‐UTR or other coding regions.^10^ In general, N^6^‐methyladenosine (m^6^A)‐modified RNAs are more attractive for RBP binding,^11^ including IGF2BPs. Thus, IGF2BPs have been identified as new m^6^A readers. Due to their sequence homology, these isoforms of IGF2BPs share 55–70% of target RNAs.^11^

As one isoform of IGF2BP, IGF2BP3 has been considered a biomarker in different types of cancer, including cervical cancer.^12^ IGF2BP3 is not expressed in normal tissue or cervical intraepithelial neoplasia (CIN) I but can be detected in 1% of CIN II, 18% of CIN III and 96% of squamous cell carcinoma (SCC) patients,^13^ indicating a close correlation with systemic malignancies. In cervical SCC, IGF2BP3 serves as a prognostic indicator, and its expression is closely associated with age, stage (FIGO) and metastasis (lymph node).^14^ IGF2BP3 overexpression results in an increase in IGF2 protein and the activation of downstream pathways, such as mitogen‐activated protein kinaseand phosphoinositide 3‐kinase , which drive cell growth.^15^, ^16^ In addition, as a m^6^ A reader, IGF2BP3 targets m^6^A‐modified RNAs, such as *MYC*, *PD‐L1*, *KPNA2* and *CDK4*, and promotes their stability and translation.^11^, ^17^, ^18^, ^19^ Many target RNAs are oncogenic proteins, and IGF2BP3 exerts an oncogenic function in cancer. Thus, as a new molecular marker, IGF2BP3 could become a potential therapeutic target for cancer treatment.

There are several molecular regulatory mechanisms regulating IGF2BP expression in cancer, including transcriptional and post‐translational regulation. At the transcriptional level, the transcription factors Nanog and NF‐κB bind to the IGF2BP3 promoter region and up‐regulate its expression for stemness maintenance and migration properties of cancer cells.^20^, ^21^ In addition, epigenetic modifications also affect the expression levels of IGF2BP3.^22^ This association has also been demonstrated in a large‐scale sequencing analysis of different cancer datasets.^23^ Several miRNAs regulate IGF2BP3 expression in different cancers, such as miRNA‐34a.^24^, ^25^ At the post‐translational level, IGF2BPs are mainly regulated by phosphorylation modification. In breast cancer, EGFR signalling regulates IGF2BP3 expression,^26^ indicating phosphorylation‐dependent regulation. Many kinases, such as mammalian target of rapamycin complex 2 (mTORC2),^27^ Src^28^ and extracellular regulated protein kinase (ERK),^29^ are able to directly phosphorylate IGF2BP1, and phosphorylation enhances the stability of its target RNAs and promotes their translation, including IGF2‐leader 3, MYC, SOX2 and β‐actin. In addition, E3 ubiquitin ligase and ubiquitin‐specific peptidase (USP) are also involved in the regulation of IGF2BP3 expression. Makorin ring finger protein (MKRN2) regulates its downstream molecules in an IGF2BP3‐dependent manner in neuroblastoma,^30^ indicating the ubiquitination modification of IGF2BP3. USP11 protects IGF2BP3 from proteasomal degradation and promotes the tumourigenesis of colorectal cancer.^31^ However, the detailed mechanism of ubiquitination modification of IGF2BP3 and its function in tumourigenesis are still unknown. We believe that dysregulation of IGF2BP3 results in abnormal accumulation of oncogenic proteins, therefore supporting the malignant state of cancer.

Interestingly, our previous mass spectrometry results showed that IGF2BPs were listed in the Parkin‐dependent ubiquitylome of cervical cancer cells, indicating that IGF2BPs are potential substrates of the E3 ubiquitin ligase Parkin. In the present study, we explored the regulation of the m^6^A reader IGF2BP3 in human cervical cancer in detail by Parkin and revealed an important role of IGF2BP3 ubiquitination in maintaining its oncogenic function. Our findings elucidate an important mechanism underlying the regulation of IGF2BP3 in cervical cancer and provide a new modulation strategy for cervical cancer treatment.

## 2 RESULTS

### 2.1 IGF2BP3 is highly expressed in human cervical cancer

**FIGURE 1:**
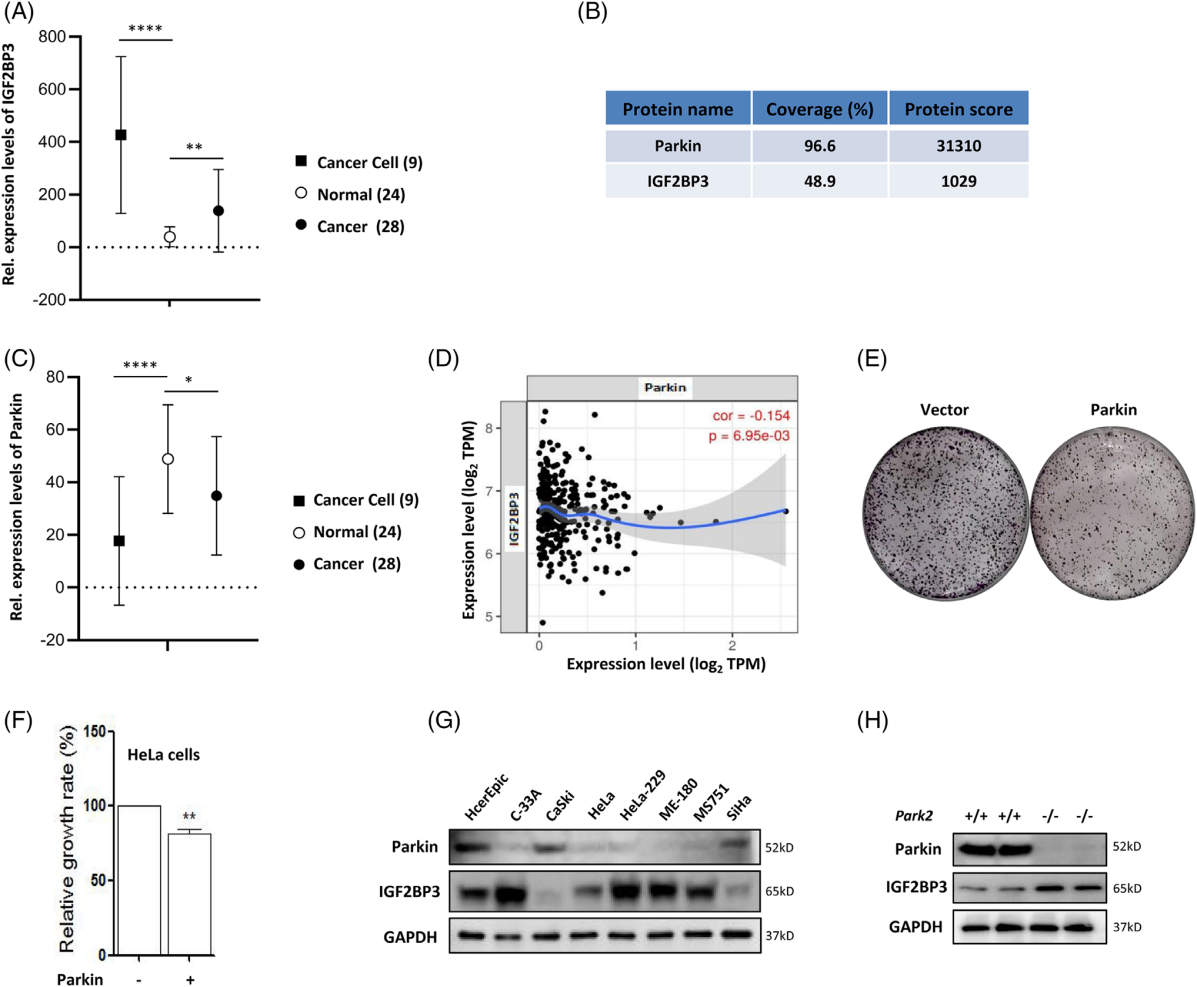
IGF2BP3 is highly expressed in human cervical cancer and negatively correlates with Parkin expression. (A) The expression levels of IGF2BP3 in a series of human cervical cancer cell lines, normal cervix and cervical cancer tissues. (B) IGF2BP3 was analysed in the Parkin pulldown complex using mass spectrometry. (C) The expression levels of Parkin in cancer cells, normal cervix and cancer tissues of patients. (D) cBioPortal analysis of the correlation between IGF2BP3 and Parkin in human cervical cancer. (E) and (F) Flag‐Parkin was overexpressed in HeLa cells; and cell growth was determined using colony formation assays. **p < 0.01. (G) The expression levels of IGF2BP3 and Parkin were analysed in human cervical cancer cells and normal cervical cells using western blotting. (H) Western blotting results showed the protein level of IGF2BP3 in the liver tissue of *Park2* ^+/+^ and *Park2* ^−/−^ mice.

Here, we analysed the expression of IGF2BPs in normal cervix epithelium (HcerEpic cells) and cervical cancer cells and tissues, including SiHa, HeLa, HeLa‐229, C‐33A, MS751 and so on. Gene Expression Omnibus (GEO) analysis demonstrated that IGF2BP3 was significantly up‐regulated in cell lines and tissues of human cervical cancer from GDS3233 (Figure 1A). In addition, GEPIA analysis showed that IGF2BP1/2/3 were up‐regulated in cervical cancer tissues (Figure S1A), but only the differential expression of IGF2BP3 was significant. Survival analysis demonstrated that IGF2BP3 had a potential association with the survival of cervical cancer patients (Figure S1B). However, more evidence is needed, and IGF2BP3 may be developed as a prognostic biomarker, accelerating the discovery of treatment targets for cervical cancer.

In our previous study,^32^ mass spectrometry results showed that IGF2BP3 may be a new substrate of Parkin (Figure 1B), and IGF2BP1/2 were also listed as its targets (Figure S1C). The Parkin protein is a tumour suppressor and exerts important functions in a variety of cellular processes implicated in tumourigenesis.^33^ GEO and GEPIA analyses also indicated the down‐regulation of Parkin in cervical cancer cells and tissues and its potential correlation with the survival of cancer patients (Figures 1C and S1D). cBioPortal analysis displayed an inverse correlation of IGF2BP3 with Parkin in human cervical cancer (Figure 1D). In HeLa human cervical cancer cells, overexpression of Parkin impaired colony formation (Figures 1E and F). Meanwhile, Parkin protein belongs to an E3 ubiquitin ligase and, cooperatively with the kinase PTEN induced putative kinase 1 (PINK1), regulates mitophagy.^34^ To validate this hypothesis, we determined IGF2BP3 expression levels in a series of cervical cancer cell lines. As shown in Figure 1G, high levels of IGF2BP3 were measured in the majority of cancer cell lines except CaSki and SiHa and the normal cell line HcerEpic. Conversely, Parkin was down‐regulated in most cervical cancer cell lines, which was opposite to the expression levels of IGF2BP3. Moreover, the liver tissue from Parkin knockout mice exhibited higher IGF2BP3 protein levels than that from wild‐type mice (Figure 1H).

### 2.2 IGF2BP3 is down‐regulated with ectopic Parkin expression

**FIGURE 2:**
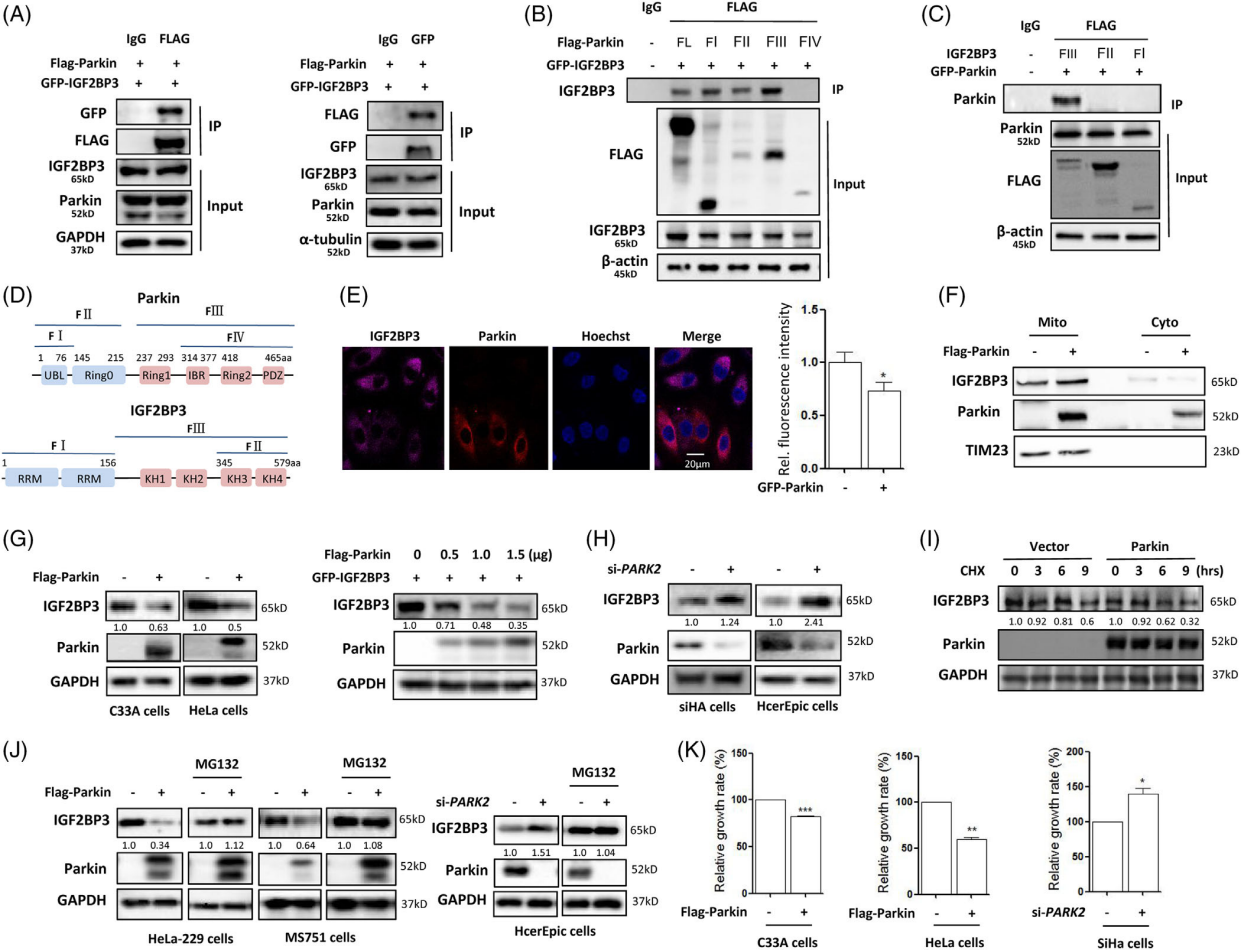
IGF2BP3 interacts with Parkin and is down‐regulated by Parkin. (A) Flag‐Parkin and GFP‐IGF2BP3 plasmids were cotransfected into HEK293 cells; and the cells were lysed. Immunoprecipitation (IP) and reverse IP analysis of the interaction of IGF2BP3 and Parkin. (B) The interaction of IGF2BP3 with different fragments of Parkin protein was analysed using IP. (FL, full length; FI1–137aa; FII1–237aa; FIII238–465aa; FIV314–465aa.) (C) The interaction of Parkin with different fragments of IGF2BP3 protein was analysed using IP. (FI1–156aa; FII345–579aa; FIII156–579aa.) (D) Diagram of different domains of Parkin and IGF2BP3. (E) Flag‐Parkin was overexpressed in HeLa cells; and the expression level of IGF2BP3 was analysed by confocal microscopy. Scale bar 10 μm. Statistical analysis was performed to analyse the fluorescence signal of IGF2BP3. (F) The expression level of IGF2BP3 in the mitochondrial fraction of HeLa cells with or without Parkin overexpression. (G) Flag‐Parkin was overexpressed in HeLa or C33A cells; and cell lysates were prepared for IGF2BP3 analysis. (H) SiHa or HcerEpic cells were transfected with siRNA specific for PARK2; and the expression of IGF2BP3 was analysed using western blotting. (I) The expression level of IGF2BP3 in Parkin‐overexpressing HeLa cells in the presence of CHX (50 μg/mL) for different time. (J) The expression level of IGF2BP3 in Parkin‐overexpressing HeLa‐229 or MS751 cells or PARK2‐knockdown HcerEpic cells under MG132 (10 μM) treatment. (K) Cell growth was determined in HeLa or C33A cells with Parkin overexpression and SiHa cells with PARK2 knockdown using CCK‐8.

To clarify the regulation of IGF2BP3 by Parkin, we performed immunoprecipitation (IP) to examine their interaction and observed that IGF2BP3 can be detected in the protein complex by Parkin pulldown (Figure 2A). Reverse IP also showed the involvement of Parkin in the protein complex of IGF2BP3 pulldown, indicating their direct interaction. Next, we sought to identify the domain of Parkin for its interaction with IGF2BP3 by expressing GFP‐IGF2BP3 and different mutants of Flag‐Parkin in HEK293 cells for IP assays, such as FI (Ubl, 1–137aa), FII (Ubl+Ring0, 1–237aa), FIII (Ring1+IBR+Ring2, 238–465aa) and FIV (IBR+Ring2, 314–465aa). As shown in Figure 2B, the domain Ubl+Ring0+Ring1 of Parkin was found to be required and sufficient for Parkin binding to IGF2BP3. Conversely, Flag‐Parkin and different deletion mutants of IGF2BP3, such as FI (1–156 aa), FII (345–579 aa) and FIII (156–579 aa), were expressed in HEK293 cells for IP assays. We found that the KH1 and KH2 domains (156–345 aa) of IGF2BP3 were required and sufficient for IGF2BP3 to interact with Parkin (Figure 2C). A diagram of the different domains of Parkin and IGF2BP3 is shown in Figure 2D.

Parkin is an E3 ubiquitin ligase and causes proteasomal degradation of mitochondrial proteins in the process of mitophagy.^34^ Since IGF2BP3 interacted with Parkin, we examined whether IGF2BP3 protein levels were also regulated by Parkin in cells. We first determined the effect of Parkin on IGF2BP3 protein levels in cells by expressing Flag‐Parkin in HeLa cells. As shown in Figure 2E, Parkin overexpression down‐regulated the pink fluorescence signal of the IGF2BP3 protein. It was also observed that pink fluorescent IGF2BP1/2 proteins were decreased with Parkin expression (Figures S2A–C). In the mitochondrial fraction, IGF2BP3 accumulated slightly with Parkin expression (Figure 2F), implying its role in the regulation of mitochondrial function. When IGF2BP3 was knocked down, the degradation of mitochondrial proteins such as COXIV and TIM23 was attenuated by Parkin (Figure S3), indicating a decrease in mitophagy levels. Consistently, the IGF2BP3 protein levels were also decreased with Parkin overexpression in HeLa and C33A cells (Figure 2G). Moreover, it displayed a dose‐dependent effect. A decrease in the expression levels of IGF2BP1 and IGF2BP2 was also detected in Parkin‐overexpressing cervical cancer cells (Figures S2D and E). Conversely, knockdown of endogenous PARK2 with small‐interfering RNA (siRNA) increased IGF2BP3 protein levels in SiHa and HcerEpic cells (Figure 2H). An increase in IGF2BP2 was also detected in HeLa‐229 cells with PARK2 knockdown (Figure S2E).

Next, we determined whether IGF2BP3 protein stability was influenced by Parkin using the protein synthesis inhibitor cycloheximide (CHX). Compared with untransfected cells, Parkin overexpression still reduced the protein levels of IGF2BP3 (Figures 2I and S2F and G), indicating its influence on IGF2BP3 protein stability. Given our finding that IGF2BP3 interacted with Parkin and was down‐regulated, we speculated that this interaction could be mediated through proteasomal degradation. Next, HeLa‐229 or MS751 cells transduced with Flag‐Parkin were treated with MG132 (a proteasome inhibitor), and IGF2BP3 levels were analysed by western blotting. As shown in Figure 2J, MG132 treatment stabilised IGF2BP3 protein levels in cells and attenuated the degradative effect of Parkin on IGF2BP3 protein. The expression levels of IGF2BP1 and IGF2BP2 were also stabilised by MG132 in HeLa cells overexpressing Parkin (Figure S2H). In HcerEpic cells with PARK2 knockdown, IGF2BP3 levels were up‐regulated, and MG132 treatment also attenuated the degradative effect of Parkin. Finally, we examined the effect of Parkin on cell proliferation and found that Parkin overexpression significantly suppressed the growth of HeLa and C33A cells, while PARK2 knockdown accelerated the growth of SiHa cells (Figure 2K), which could be associated with the expression levels of IGF2BP3.

### 2.3 IGF2BP3 is ubiquitinated by Parkin in human cervical cancer

**FIGURE 3:**
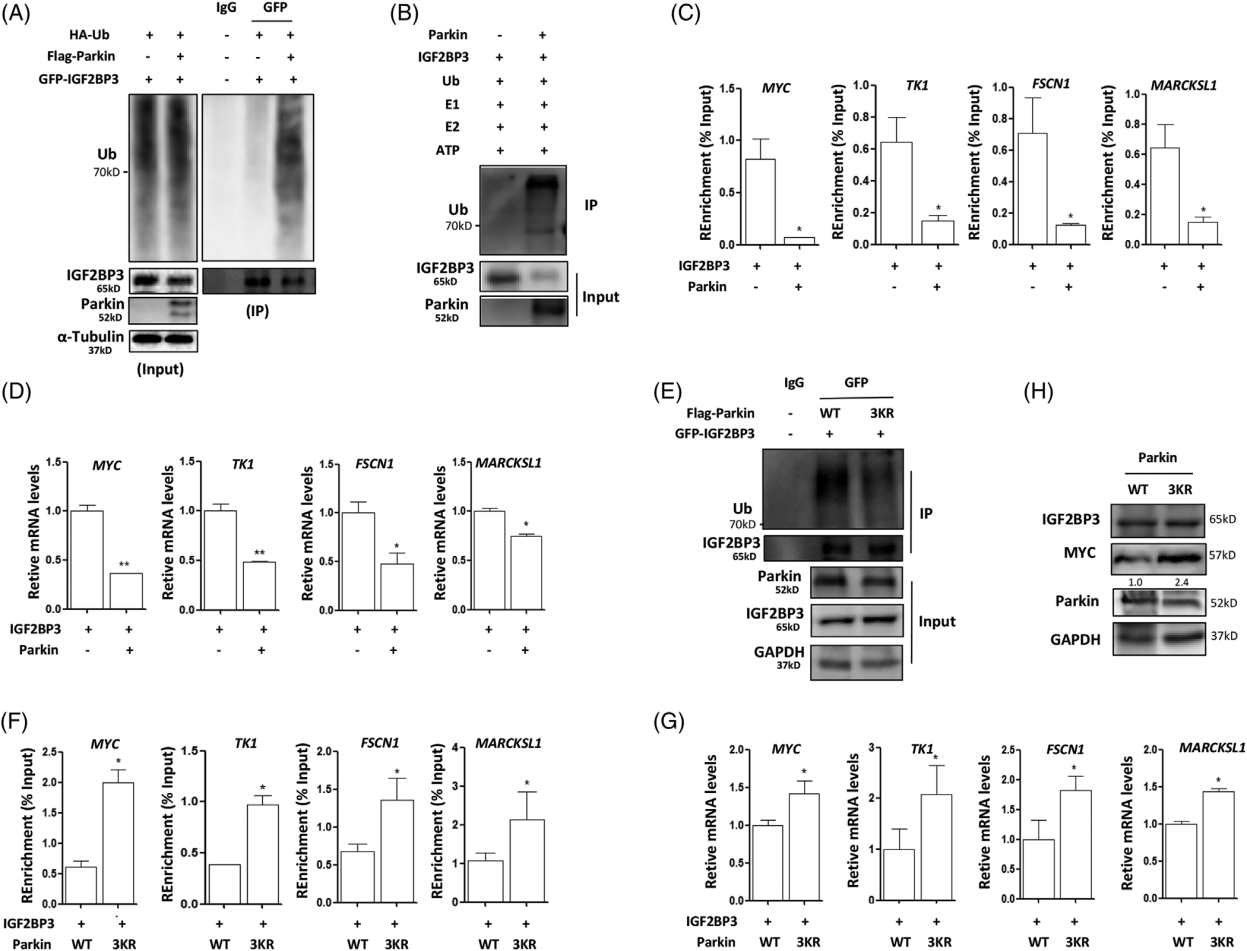
IGF2BP3 is ubiquitinated by Parkin in cervical cancer cells. (A) In vivo ubiquitination assay. GFP‐IGF2BP3, Flag‐Parkin and HA‐ubiquitin plasmids were cotransfected into HeLa cells; and the cells were lysed for IP. The level of ubiquitin was detected in the protein complex by GFP pulldown. (B) In vitro ubiquitination assay. The purified IGF2BP3 protein reacted with the purified Parkin protein in the presence of E1, E2, ubiquitin and ATP. Western blotting was applied to examine the levels of ubiquitin. (C) GFP‐IGF2BP3 plasmid was transfected into HeLa cells alone or with Flag‐Parkin; and cells were collected for RNA‐binding protein IP. The levels of target RNAs of IGF2BP3 were determined using PCR. (D) As in C, cells were harvested for RNA extraction; and real‐time PCR was performed to measure the levels of the indicated genes. (E) GFP‐IGF2BP3 plasmid was transfected into HeLa cells together with Flag‐Parkin or its mutant; and cells were lysed for IP. The level of Parkin was determined in the protein complex by GFP pulldown. (F) HeLa cells were cotransfected with GFP‐IGF2BP3 and wild‐type or mutant Parkin; and the cells were collected for RNA‐binding protein IP. The levels of target RNAs of IGF2BP3 were examined using PCR. (G) As in F, RNA extraction was performed in the treated cells; and the levels of the indicated genes were detected using PCR. (H) As in F, western blotting was used to measure the MYC protein level.

To determine whether IGF2BP3 was ubiquitinated by Parkin, GFP‐IGF2BP3, Flag‐Parkin and HA‐ubiquitin were overexpressed in HeLa cells for an in vivo ubiquitination assay. Parkin overexpression enhanced IGF2BP3 ubiquitination levels in cells (Figure 3A). Moreover, it proved to be K48‐linked ubiquitination (Figure S4A). Similarly, the ubiquitination level of IGF2BP1 was up‐regulated by Parkin (Figure S4B). An in vitro ubiquitination assay was also performed to determine the effect of Parkin on IGF2BP3 ubiquitination by using recombinant Parkin and IGF2BP3 proteins. Indeed, the presence of Parkin protein resulted in the ubiquitination of IGF2BP3 in vitro (Figure 3B).

IGF2BP3 plays oncogenic roles in cancers as a m^6^A reader.^11^ With Parkin expression, we observed that the binding of IGF2BP3 to its target genes, including MYC, TK1, FSCN1 and MARCKSL1, was significantly attenuated (Figure 3C), indicating the decreased function of IGF2BP3. The target gene expression levels were significantly down‐regulated with Parkin overexpression (Figure 3D), which could be associated with the decrease of IGF2BP3 function. However, Parkin mutation (a mutant Parkin with impaired ubiquitination activity)^32^ resulted in a decrease in IGF2BP3 ubiquitination levels in cells under normal conditions (Figure 3E).

SAHA is an HDAC inhibitor and can cause the acetylation of Parkin and enhance its function. Under SAHA treatment, endogenous IGF2BP3 levels were also decreased in HeLa cells overexpressing Parkin (Figure S5A). Meanwhile, the binding of Parkin to IGF2BP3 was also abolished with Parkin mutation under treatment with SAHA (Figure S5B). With the ubiquitination activity of Parkin impairment, the function of IGF2BP3 was also altered accordingly. As shown in Figures 3F and S5C, with the Parkin mutation, the binding of IGF2BP3 to its target genes, such as MYC, TK1, FSCN1 and MARCKSL1, was also significantly increased. In mutant Parkin‐expressing cells, the expression levels of IGF2BP3 target genes were significantly up‐regulated compared with those in wild‐type Parkin‐expressing cells (Figures 3G and S5D). In addition, western blotting results also showed that MYC protein levels were increased with Parkin mutation (Figure 3H). Taken together, these results suggest that IGF2BP3 is regulated by the ubiquitination activity of Parkin, which could affect its oncogenic function.

### 2.4 Identification and validation of the ubiquitination site of IGF2BP3

**FIGURE 4:**
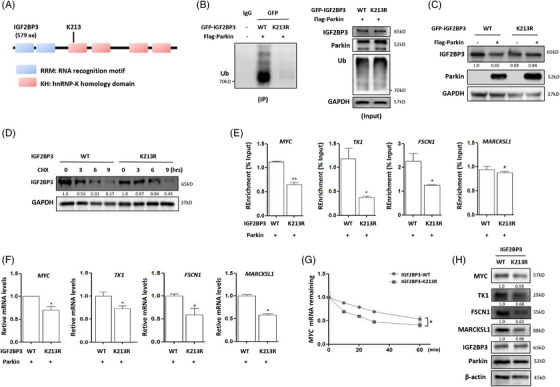
The ubiquitination site of IGF2BP3 was identified at K213. (A) The protein domain of human IGF2BP3 and its ubiquitination site. (B) The ubiquitination level of IGF2BP3 was determined using IP. Flag‐Parkin and HA‐ubiquitin plasmids were cotransfected into HeLa cells with wild‐type IGF2BP3 or mutant IGF2BP3; and the cells were lysed for GFP pulldown. (C) The protein levels of IGF2BP3 were measured using western blotting in wild‐type or mutant IGF2BP3‐overexpressing cells with or without Parkin. (D) The protein level of IGF2BP3 in wild‐type or mutant IGF2BP3‐overexpressing HeLa‐Parkin cells in the presence of CHX (50 μg/mL) for different time. (E) The binding of IGF2BP3 to its target RNAs was determined using RNA‐binding protein IP. HeLa‐Parkin cells were overexpressed with wild‐type or mutant IGF2BP3; and PCR was applied to evaluate the levels of target RNAs in pulldown RNA by IGF2BP3. (F) As in E, the levels of target RNAs of IGF2BP3 were measured using PCR in HeLa‐Parkin cells transfected with wild‐type or mutant IGF2BP3. *p < 0.05 G. As in E, the mRNA level of MYC gene in cells was determined using real‐time PCR under treatment with actinomycin D (5 μg/mL) for different time. (H) As in E, the expression levels of MYC, TK1, FSCN, MARCKSL1 proteins were examined by western blotting.

To identify the ubiquitination sites in IGF2BP3 modified by Parkin, LC–MS/MS was performed to analyse the immunoprecipitated IGF2BP3 proteins from Parkin‐overexpressing HeLa cells. The lysine at 213 of IGF2BP3 (K213) was found to be the candidate ubiquitination site, and its spectrum image was drawn and is shown in Figure S6 and Table S1, which is located in the first KH‐domain of IGF2BP3 (Figure 4A). The lysine at this site of GFP‐IGF2BP3 was mutated to arginine (K213R), and whether this mutation affected IGF2BP3 ubiquitination was examined using an in vivo ubiquitination assay. Compared with wild‐type IGF2BP3, the K213R mutation dramatically abolished the ability of Parkin to degrade IGF2BP3 (Figure 4B). Consistently, in mutant IGF2BP3‐expressing cells, Parkin failed to down‐regulate the protein levels of IGF2BP3 (Figures 4C and S7A). Next, we determined IGF2BP3 protein stability using the protein synthesis inhibitor CHX. Compared with wild‐type IGF2BP3, the K213 mutation delayed the degradation of IGF2BP3 (Figure 4D), indicating its influence on IGF2BP3 protein stability.

m^6^A is the most prevalent internal modification of eukaryotic mRNA. IGF2BP3 is a family of m^6^A readers that recruits cofactor proteins to stabilise m^6^A‐modified transcripts.^11^ Because the ubiquitination site of IGF2BP3 is in the GXXG motif of the KH1 domain, its mutation may have profound consequences on its target RNA binding. Indeed, IGF2BP3 binding to its target genes, including MYC, TK1 and FSCN1, was markedly attenuated with its mutation (Figures 4E and S7B). In mutant IGF3BP3‐expressing cells, the expression levels of IGF2BP3 target genes were also significantly down‐regulated compared with those in wild‐type IGF2BP3‐expressing cells, either under normal conditions or after treatment with the HDACi SAHA (Figures 4F and S7C and D). Under treatment with actinomycin D, which inhibits DNA‐dependent RNA polymerase activity, the remaining mRNA levels of MYC, TK1 and FSCN1 were lower in mutant IGF3BP3‐expressing cells (Figures 4G and S7E), indicating the attenuated mRNA stability of IGF2BP3 target genes. In addition, the results of western blotting also showed that IGF2BP3 mutation resulted in a decrease in MYC, TK1, FSCN1, MARCKSL1 protein levels (Figure 4H). In addition, we performed dot blot analysis of m^6^A‐modified RNA levels using a specific m^6^A antibody and found that with IGF2BP3 mutation, the m^6^A RNA levels were decreased in HeLa‐Parkin cells, indicating the weakened function of IGF2BP3 (Figure S7F). However, whether m^6^A modification mediates the down‐regulation of IGF2BP3 target genes still needs more exploration. Taken together, these results demonstrate that IGF2BP3 ubiquitination is indispensable for its oncogenic function.

### 2.5 Identification of the targets of IGF2BP3 through transcriptome sequencing

**FIGURE 5:**
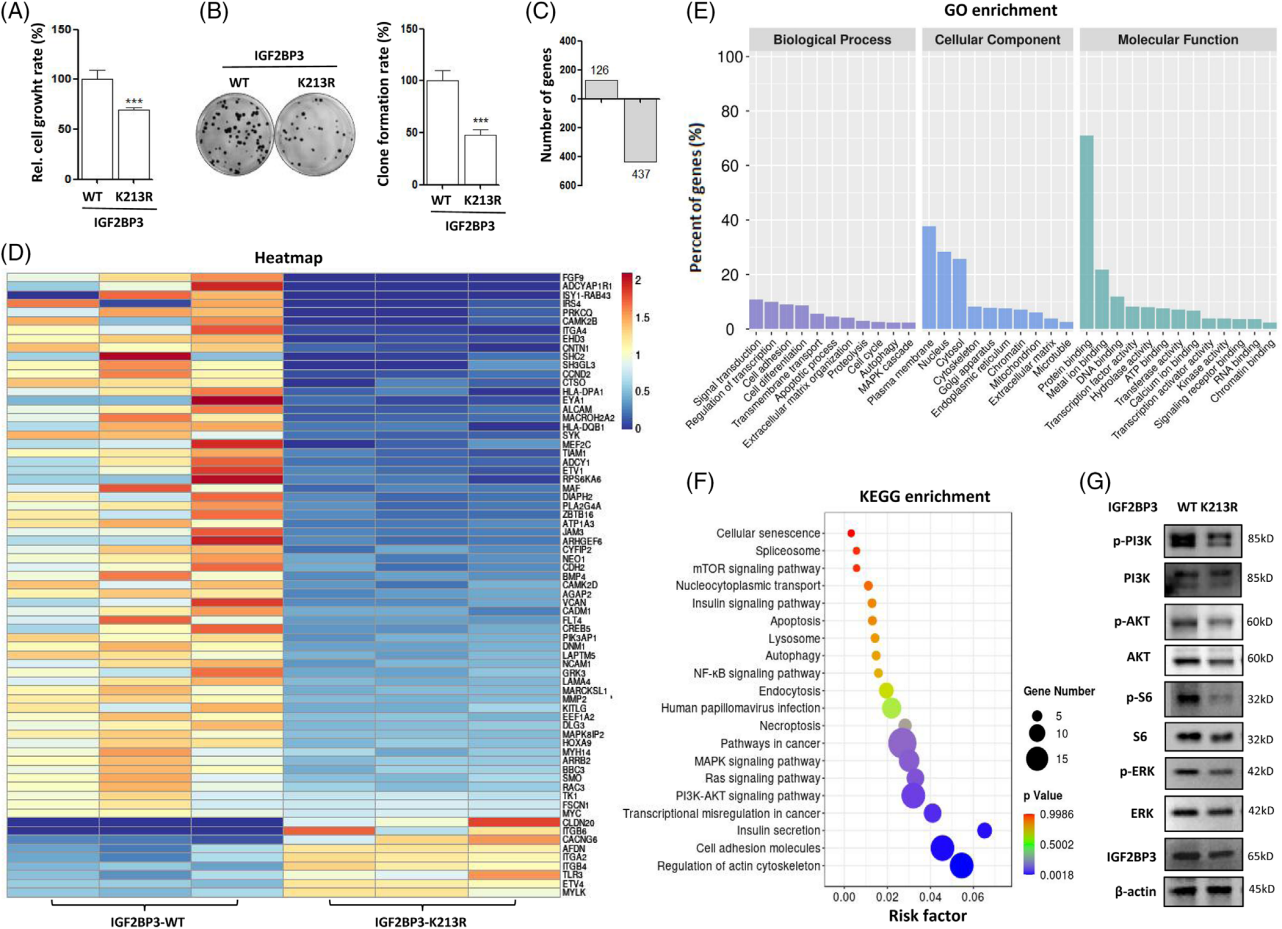
Transcriptome analysis of the target RNAs of IGF2BP3 in human cervical cancer. (A) HeLa‐Parkin cells were overexpressed with wild‐type or mutant IGF2BP3. The cell growth rate was measured using the CCK‐8 assay and statistically analysed. (B) Analysis of IGF2BP3 ubiquitination on cell growth by colony formation. (C) RNA extraction was conducted from cells and used for transcriptome sequencing. The expression of differentially expressed genes was analysed in the mutant and wild‐type IGF2BP3 groups. (D) Heatmap of the most differential gene expression profiles for wild‐type and mutant IGF2BP3‐expressing cells. The up‐regulated or down‐regulated genes are marked in red or blue, respectively. (E) GO analysis of biological process, cellular localisation and molecular function of the most differential targets of IGF2BP3. (F) KEGG enrichment of the most differential signalling pathways in wild‐type or mutant IGF2BP3‐expressing cells. The dot represents the gene number. Red and blue represent *p* values. (G) Western blotting analysis of the phosphorylation levels of PI3K, AKT, S6 and ERK in wild‐type or mutant IGF2BP3‐expressing cells.

The above results demonstrated that IGF2BP3 mutation attenuated its oncogenic function. Consistently, the CCK‐8 assay results showed that IGF2BP3 mutation significantly inhibited cervical cancer cell growth (Figure 5A). In addition, colony formation assay results also showed that the number of colonies formed was obviously decreased with IGF2BP3 mutation (Figure 5B). To clarify the associated molecules and signalling pathways, we performed transcriptome sequencing to identify potential targets of IGF2BP3. As shown in Figure 5C, numerous genes were screened in wild‐type or mutant IGF2BP3‐overexpressing cells. Compared with the wild‐type IGF2BP3 group, 437 genes were down‐regulated, and only 126 genes were up‐regulated in the mutant IGF2BP3 group. A heatmap was generated using the most differentially expressed genes in the mutant IGF2BP3 group compared to the wild‐type IGF2BP3 control group (Figure 5D). They displayed different transcriptional profiles, and the IGF2BP3 mutation was noticeably distinct from the IGF2BP3 wild‐type control. The down‐regulation of a series of genes was associated with cell proliferation pathways, including FGF9, SHC2, SH3GL3, IRS4, PRKCQ, ITGA4, SYK, PRS6KA6, PIK3AP1, MAPK8IP2 and GRK3. Among them, target RNAs of IGF2BP3 were also listed, such as MYC, TK1, FSCN1 and MARCKSL1, indicating the inhibition of IGF2BP3 function.

The most differentially expressed target genes of IGF2BP3 were subjected to GO analysis. Cellular component analysis showed that these targets belong to different cellular parts (Figure 5E) and are mainly enriched in the plasma membrane, nucleus, cytosol and so on. These targets participate in various biological processes, especially in cell growth, such as the regulation of transcription, cell adhesion, cell differentiation and the cell cycle. Molecular functions involve the regulation of transcription, such as DNA, RNA binding, transcription factor or activator activity (Figure 5E). In addition, these target genes also exert their function in signal transduction and the binding activity of proteins, metal ions, signalling receptors and so on.

Kyoto Encyclopedia of Genes and Genomes (KEGG) analysis showed that the most differentially expressed targets of IGF2BP3 were mainly associated with the insulin signalling pathway (Figure 5F), including downstream PI3K/AKT/mTOR signalling and RAS/RAF/MEK/ERK signalling. IGF2BP3 is an RBP that binds to the UTR of IGF‐2 mRNA, thereby activating its translation. Thus, IGF2BP3 is regarded as a pro‐proliferative and pro‐invasive marker that activates oncogenic MAPK and PI3K pathways through the regulation of IGF‐2.^35^ With IGF2BP3 mutation, these cell proliferation pathways were significantly inhibited with the down‐regulation of related genes (Figure 5D). Other signalling pathways were also dysregulated, including apoptosis, autophagy, necroptosis, lysosome, endocytosis and so on. Western blotting results also showed a decrease in phosphorylated PI3K, AKT, S6 and ERK (Figure 5G), indicating the inhibition of the mTOR and ERK signalling pathways.

### 2.6 The regulation of Parkin‐dependent mitophagy by IGF2BP3 ubiquitination

**FIGURE 6:**
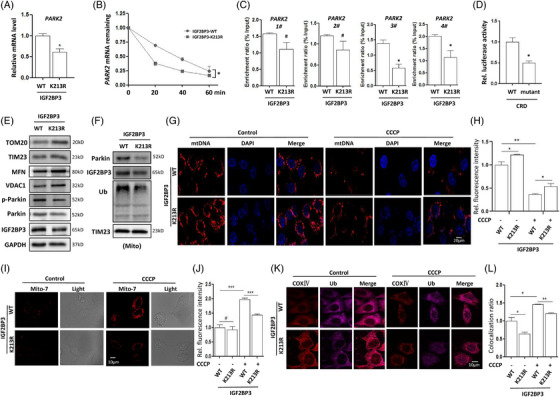
The negative regulatory role of IGF2BP3 in Parkin‐mediated mitophagy. (A) Wild‐type or mutant IGF2BP3 plasmids were transfected into HeLa‐Parkin cells; and RNA extraction was conducted. PCR was used to measure the expression level of the PARK2 gene. (B) As in A, the expression level of the PARK2 gene in cells was examined after treatment with actinomycin D (5 μg/mL) for different time using PCR. (C) The binding of IGF2BP3 to the PARK2 gene was determined using RNA‐binding protein IP. HeLa‐Parkin cells were transfected with IGF2BP3; and PCR was applied to examine the levels of PARK2 in RNA pulldown by IGF2BP3. (D) A luciferase reporter assay was applied to examine the binding of IGF2BP3 to the wild‐type or mutant CRD of the PARK2 gene. (E) As in A, cells were collected for western blotting analysis of phosphorylated Parkin and mitochondrial protein levels. (F) The expression levels of Parkin and ubiquitin were determined in the mitochondrial fraction using western blotting. (G‐H) As in A, cells were first treated with CCCP and incubated with mtDNA antibodies. Cell fluorescence was detected by confocal microscopy. Scale bar 10 μm. The fluorescent signal was examined using ImageJ and statistically analysed. (I‐J) The mKeima‐Red‐Mito‐7 plasmid was transfected into HeLa‐Parkin cells; and the cells were treated with CCCP (10 μM; 2 h). Confocal microscopy was applied to detect cell fluorescence. Excitation: 440 nm; emission: 620 nm. Scale bar 10 μm.The fluorescent signal of Mito‐7 was examined using ImageJ and statistically analysed. (K‐L) As in A, cells were first treated with CCCP. After fixation, the cells were incubated with COXIV and ubiquitin antibodies; and cell fluorescence was detected by confocal microscopy. Scale bar 10 μm. The co‐localisation of COXIV and ubiquitin was analysed using ImageJ and statistical analysis was performed.

The above results demonstrated the regulation of Parkin on its downstream molecule IGF2BP3 through ubiquitination. Conversely, we examined whether the RBP IGF2BP3 exerts a regulatory effect on Parkin. In cells, PINK1 and Parkin work cooperatively to eliminate damaged mitochondria through the autophagy pathway.^34^ Here, we first determined the expression levels of Parkin in wild‐type or mutant IGF2BP3‐expressing cells. As shown in Figure 6A, the IGF2BP3 mutation significantly down‐regulated the mRNA levels of PARK2 in HeLa‐Parkin cells. Under actinomycin D treatment, the mRNA stability of PARK2 was also attenuated with IGF2BP3 mutation, which resulted in lower levels of remaining PARK2 mRNA (Figure 6B). Next, we examined the binding activity of IGF2BP3 to the PARK2 gene. IGF2BP3 preferentially binds to the consensus region with a ‘GGAC’ m^6^A core motif, termed the coding region instability determinant (CRD).^11^ Accordingly, we designed four pairs of primers to detect their binding at the 3′UTR of the PARK2 gene, and those candidate binding sites are marked in red (Figure S8). As expected, IGF2BP3 binding to the PARK2 gene was markedly attenuated in the region of primers #3 and #4 (Figure 6C), which may be the reason for the down‐regulation of PARK2 mRNA levels. With the mutation of CRD at the 3′UTR of the PARK2 gene, the binding activity of IGF2BP3 to the PARK2 gene was significantly down‐regulated (Figure 6D). Finally, we examined the level of mitophagy in HeLa‐Parkin cells. The results of western blotting showed that IGF2BP3 mutation resulted in an increase in mitochondrial protein levels (Figure 6E), such as TOM20, TIM23, VDAC1 and MFN, indicating the inhibition of mitophagy. Meanwhile, the levels of phosphorylated Parkin were decreased with IGF2BP3 mutation. In the mitochondrial fraction, IGF2BP3 mutation led to less Parkin protein translocation and lower ubiquitination levels of mitochondrial proteins (Figure 6F). The degradation of mtDNA was also determined and more mtDNA was detected in mutant IGF2BP3‐expressing cells (Figure 6G‐H); in response to CCCP treatment, the degradation of mtDNA was alto attenuated due to IGF2BP3 mutation. To examine the occurrence of mitophagy, cells were transfected with mito‐Keima to determine mitochondrial movement into lysosomes.^36^ As shown in Figures 6I and J, fewer red spots were detected in the cytoplasm of mutant IGF2BP3‐expressing cells than in that of wild‐type IGF2BP3‐expressing cells in response to CCCP treatment, indicating the formation of fewer mitolysosomes. In addition, confocal imaging results also displayed a stronger fluorescent signal of COXIV and less co‐localisation of COXIV with ubiquitin proteins with the IGF2BP3 mutation, either under normal conditions or after CCCP treatment (Figures 6K and L). Collectively, the above results demonstrated that IGF2BP3 ubiquitination is required for Parkin‐mediated mitophagy. In addition, the role of mitophagy in the oncogenic function of IGF2BP3 was also determined. As shown in Figure S9, IGF2BP3 overexpression promoted the growth of SiHa cells, but this effect was significantly attenuated by PINK1 knockdown, suggesting that mitophagy promotes cell survival. Thus, cell growth inhibition by IGF2BP3 mutation may be attributed to a decrease in mitophagy levels.

### 2.7 IGF2BP3 mutation attenuates the tumourigenesis of cervical cancer

**FIGURE 7:**
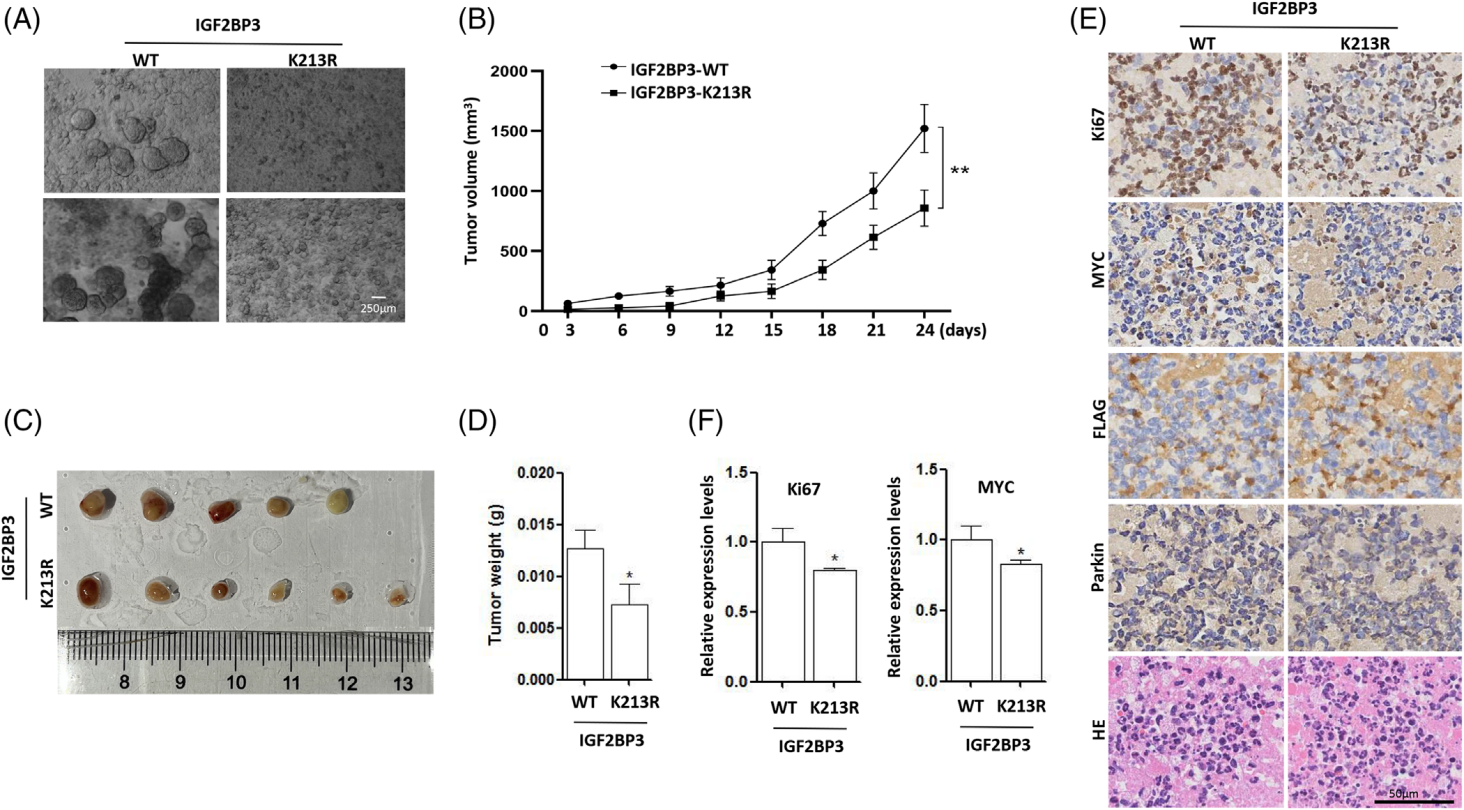
Mutation of IGF2BP3 attenuates the tumourigenesis of cervical cancer. (A) Representative images of tumour spheroids formation from HeLa‐Parkin cells expressing wild‐type or mutant IGF2BP3. Scale bar 250 μm. (B) Nude mice were subcutaneously injected with HeLa‐Parkin cells expressing wild‐type or mutant IGF2BP3 to establish a xenograft tumour model. Xenograft tumour volumes were measured twice per week and then quantified and statistically analysed. **p < 0.01. (C) Representative images of the xenograft tumours. (D) The excised tumours in the two groups were weighed and statistically analysed. (E) IHC staining of Ki‐67, MYC, FLAG and Parkin and H&E staining were performed on serial sections of tumour tissues in the two groups. (F) The protein levels of Ki‐67 and MYC in tumour tissues were analysed using ImageJ; and statistical analysis was performed.

Finally, the effect of IGF2BP3 ubiquitination on cervical cancer growth was determined using tumour spheroids formation. As shown in Figure 7A, HeLa‐Parkin cells expressing wild‐type IGF2BP3 formed a large number of tumour spheroids, but it was hardly observed in mutant IGF2BP3‐expressing cells. In addition, a xenograft tumour model was established to further evaluate the effect of IGF2BP3 ubiquitination on tumourigenesis. Nude mice were subcutaneously injected with sufficient cervical cancer cells, including HeLa‐Parkin cells with wild‐type or mutant IGF2BP3 expression. As shown in Figure 7B, IGF2BP3 mutation resulted in a significant tumour suppression when compared with wild‐type IGF2BP3 group. Two weeks later, tumour‐bearing mice were sacrificed, and tumours were excised from mice and weighed. The IGF2BP3 wild‐type and mutant groups displayed a significant difference in tumour volume and weight with time (Figures 7C and D), indicating decreased tumourigenesis with IGF2BP3 mutation. Tumour tissues were routinely fixed with buffered 4% paraformaldehyde and used for histopathological and immunohistochemical analysis. As shown in Figures 7E and F, IGF2BP3 mutation significantly decreased the expression levels of Ki‐67 and the oncogenic protein MYC, indicating a decrease in cell proliferation. H&E staining results also showed that the mutant IGF2BP3 group displayed fewer cancer cells, smaller cell sizes and accumulated chromatin condensation compared with the wild‐type IGF2BP3 group. The above results demonstrate that IGF2BP3 ubiquitination is indispensable for its oncogenic function in cervical cancer.

**FIGURE 8:**
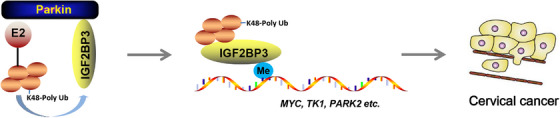
A schematic model of IGF2BP3 ubiquitination and its important role in tumourigenesis.

The molecular mechanism controlling IGF2BP3 ubiquitination is described in detail and summarised in Figure 8. The RBP IGF2BP3 was ubiquitinated by the E3 ubiquitin ligase Parkin in human cervical cancer, which occurred at K213 in the first KH domain of IGF2BP3. Ubiquitination modification plays an important role in maintaining the oncogenic function of IGF2BP3. IGF2BP3 mutation resulted in the loss of its function in the activation of oncogenic PI3K and MAPK signalling pathways, including mitophagy, which resulted in the inhibition of cervical cancer cell growth and tumourigenesis.

## 3 DISCUSSION

IGF2BP3 is an RBP and exerts oncogenic function in cancers as a new m^6^A reader.^11^ IGF2BP3 has a high correlation with human cervical SCC and is regarded as a prognostic indicator.^14^ Here, we also examined the mRNA levels of IGF2BP3 in human cervical cancer tissues but found no significant association with the survival of cancer patients. The expression of IGF2BPs can be regulated at different levels as previously described. At the post‐translational level, IGF2BPs are mainly regulated by different kinases through phosphorylation.^29^, ^37^ With phosphorylation, they promote the translation of their target RNAs and enhance their oncogenic function. In addition, some deubiquitinases and E3 ubiquitin ligases are also reported to be involved in the regulation of IGF2BPs.^30^, ^38^ However, a more detailed mechanism is still unknown. In this study, we investigated the regulation of IGF2BP3 by the E3 ubiquitin ligase Parkin and identified its ubiquitination site in human cervical cancer. It was revealed that IGF2BP3 ubiquitination by Parkin is a critical mechanism in maintaining the oncogenic function of IGF2BP3. Thus, in future studies, we will analyse the ubiquitination level of IGF2BP3 in human cervical cancer tissues and its association with the prognosis of cancer patients. We believe that IGF2BP3 ubiquitination could be a good biomarker for cervical cancer.

Ubiquitination, one form of post‐translational modification, is processed by the ubiquitin–proteasome system (UPS), and ubiquitin is conjugated to a substrate protein, which regulates protein stability and localisation.^39^ This process involves 3 distinct enzymes, including ubiquitin activating, conjugating and ligation enzymes.^40^ Among them, ubiquitin ligase E3 is crucial in the UPS, which determines substrate selectivity.^41^ IGF2BP3 is an oncogenic protein in a number of malignancies,^14^ including cervical SCC. Moreover, it has been revealed as a prognostic indicator of cervical SCC, and high levels of IGF2BP3 correlate with poor survival.^14^ However, it remains unknown whether the oncogenic activity of IGF2BP3 is related to its ubiquitination in cervical cancer. Here, mass spectrometry analysis showed that the E3 ubiquitin ligase Parkin promoted IGF2BP3 polyubiquitination in cervical cancer (Figures 3 and 4). IGF2BP1 and IGF2BP2 are also regulated by Parkin, but we did not explore their ubiquitination modification or their role in the tumourigenesis of cervical cancer in the current study. A ubiquitination assay demonstrated that mutation of IGF2BP3 at K213 could block Parkin‐mediated ubiquitination, reduce the oncogenic function of IGF2BP3 and inhibit cervical cancer cell proliferation (Figures 4 and 5). Therefore, the ubiquitination of IGF2BP3 is positively related to its activity in carcinogenesis. In a previous study,^30^ IGF2BP3 was reported to be regulated by the E3 ubiquitin ligase MKRN2, which is also a tumour suppressor. In neuroblastoma, MKRN2 down‐regulated the expression of IGF2BP3‐targeted downstream pathways, indicating a negative regulatory effect of ubiquitination on IGF2BP3 function. Our findings were contradictory to the above study, which may be associated with cancer type. In addition to Parkin, our mass spectrometry results also showed that different E3 ubiquitin ligases were identified in the IGF2BP3 pulldown complex, including HUWE1, UBRs, RNFs, ARIH1, HECTD1, TRIP12 and TRIMs. We speculate that the association of IGF2BP3 ubiquitination with its oncogenic function may be cooperatively regulated by some E3 ubiquitin ligases to some extent.

Mitophagy is a type of selective autophagy and serves to maintain mitochondrial homeostasis by eliminating damaged mitochondria.^42^ In mammalian cells, the kinase PINK1 and the E3 ubiquitin ligase Parkin cooperatively regulate the disposal of damaged mitochondria through the autophagy pathway.^34^ Many mitochondrial membrane proteins are downstream molecules of Parkin.^43^ Mitophagy receptors recognise these ubiquitinated proteins and guide them in forming mitophagosomes. In our study, we identified IGF2BP3 as a new substrate of Parkin, and it also participated in the process of mitophagy. In Parkin‐mediated mitophagy, knockdown of IGF2BP3 attenuated the levels of mitophagy (Figure S3). In HeLa cells, IGF2BP3 was ubiquitinated and translocated into mitochondria with Parkin (Figures 3A and B and 2F). IGF2BP3 mutation delayed its degradation by Parkin and enhanced protein stability (Figures 4C and D). Conversely, IGF2BP3 also exerted a positive regulatory role in Parkin function. For example, as shown in Figure 6, IGF2BP3 mutation attenuated the level of Parkin‐mediated mitophagy due to a decrease in the mRNA stability of the PARK2 gene. It may be responsible for cervical cancer cell growth inhibition with IGF2BP3 mutation. There is increasing evidence^44^ showing that excessive mitophagy accelerates a number of pathologies, including cancer. A correlation between mitophagy and enhanced cancer development has been observed, leading to the emerging concept that mitophagy in general is a tumour promotion mechanism. However, more studies are needed to clarify the function of mitophagy in the tumourigenesis of IGF2BP3.

In summary, we identified the ubiquitination site of IGF2BP3 and revealed its importance in regulating IGF2BP3 function, which is an important mechanism for IGF2BP3 in tumourigenesis. The ubiquitination modification of IGF2BP3 is able to preserve the activation of cell proliferation pathways, including mitophagy. IGF2BP3 is a critical RNA regulator responsible for modulating post‐transcriptional events in the cell. The post‐translational regulation of IGF2BP3 also provides new insights into epigenetic changes in cancer.

## 4 EXPERIMENTAL

### 4.1 Cell lines

Normal cervical cell line HcerEpic and human cervical cancer cell lines, including CaSki, C‐33A HeLa, HeLa‐229, ME‐180, MS751 and SiHa, were obtained from National Collection of Authenticated Cell Cultures of China. All cell lines were maintained in DMEM (E600003; Sangon Biotech) containing 10% foetal bovine serum (E510008; Sangon Biotech) in a 5% CO_2_ atmosphere at 37°C.

### 4.2 Reagents

Antibodies were obtained as follows: α‐tubulin (Cat No. AF5012; Beyotime), TIM23 (Cat No. sc‐514463; Santa Cruz Biotechnology), GFP‐Trap^®^ Agarose (Cat No. gta; ChromoTek), anti‐FLAG^®^ M2 affinity gel (Cat No. A2220; Sigma–Aldrich), m^6^A (Cat. No. 56593; CST), TOM20 (Cat No. 42406; CST) and COXIV (Cat No. 11967; CST).

All of these antibodies were purchased from Proteintech: glyceraldehyde‐3‐phosphate dehydrogenase (GAPDH) (Cat No. 60004‐1‐Ig), FLAG (Cat No. 20543‐1‐AP), FSCN1 (Cat No. 14384‐1‐AP), GFP (Cat No. 50430‐2‐AP), IGF2BP1 (Cat No. 22803‐1‐AP), IGF2BP2 (Cat No. 11601‐1‐AP), IGF2BP3 (Cat No. 14642‐1‐AP), Ki67 (Cat No. 27309‐1‐AP), MYC (Cat No. 10828‐1‐AP), MARCKSL1 (Cat No. 10004‐2‐Ig), LC3 (Cat No. 14600‐1‐AP), Parkin (Cat No. 14060‐1‐AP), TK1 (Cat No. 15691‐1‐AP), ubiquitin (Cat No. 10201‐2‐AP), VDAC1 (Cat No. 10866‐1‐AP).

Recombinant proteins were purchased from Novus: ubiquitin‐activating Enzyme/UBE1 (Cat No. E‐305), UbcH7/UBE2L3 (Cat No. E2‐640), ubiquitin (Cat No. U‐100H), Parkin (Novus, E3‐160). Recombinant IGF2BP3 protein was purchased from Abnova (Cat No. H00010643‐P01).

All chemicals were purchased from MedChemExpress: actinomycin D (Cat No. HY‐17559), CCCP (Cat No. HY‐100941), MG132 (Cat No. HY‐13259), CHX (Cat No. HY‐12320), SAHA (Cat No. HY‐10221).

### 4.3 siRNA and plasmids transient transfection

The scrambled RNAi oligonucleotides and siRNAs targeting PARK2 or IGF2BP3 (GenePharma) were transfected into cells using the Lipo8000™ (C0533; Beyotime) according to the manufacturer's protocol. After 48 h, the cells were subjected to the designated treatment. For plasmid transfection, cells were transiently transfected with GFP‐IGF2BP3, Flag‐Parkin or HA‐ubiquitin plasmids using the Lipo8000™ according to the manufacturer's protocol. Some plasmids were kindly provided by Prof. Shen Han‐Ming (University of Macau, China) as described.

### 4.4 In vivo ubiquitination assay

Cells were co‐transfected with GFP‐IGF2BP3, Flag‐Parkin and HA‐ubiquitin for 24 h. Cells were then treated with MG132 (10 μM) for 8 h and the levels of IGF2BP3 ubiquitination was determined by GFP‐Trap^®^ Agarose pulldown followed by western blot analysis.

### 4.5 In vitro ubiquitination assay

Reaction mixtures consisted of buffer (5 mM MgCl_2_, 50 mM Tris, pH 7.4, 1 mM DTT, 2 mM ATP), 0.5 μg ubiquitin‐activating enzyme/UBE1, 0.5 μg UbcH7/UBE2L3, 5 μg ubiquitin, 0.4 μg purified HIS‐IGF2BP3 protein and 0.5 μg recombinant Parkin protein. After incubation for 3 h at 37°C, post‐reaction mixtures were used for HIS tag pulldown and western blot analysis with ubiquitin antibody.

### 4.6 Mitochondria isolation and analysis

After indicated treatment, cells were harvested, resuspended in mitochondria isolation reagent and transferred to Dounce Tissue Grinder. After homogenisation, cellular mitochondria were isolated according to the manufacture's description (C3601; Beyotime). The isolated mitochondria were lysed with western buffer (P0013; Beyotime). The sample was separated on 12% (w/v) SDS‐PAGE gels through electrophoresis.

### 4.7 Western blotting

After the indicated time of designated treatment, cells were collected and rinsed with PBS. The whole cell lysates were prepared in the protein lysis buffer (P0013; Beyotime) with proteinase inhibitor. An equal amount of protein was resolved by SDS‐PAGE and transferred onto PVDF membrane. After blocking with 5% non‐fat milk, the membrane was probed with designated primary and secondary antibodies, developed with BeyoECL Star (P0018AS; Beyotime) and visualised using ChemiDoc MP Imaging System (JS‐1075; P&Q Science&Technology).

### 4.8 IP assay

Briefly, the cells were lysed on ice for 30 min with the IP buffer (P0013; Beyotime) with phosphatase and protease inhibitor. Cell lysates were incubated with indicated antibodies overnight with gentle rocking at 4°C. The immunoprecipitates were then washed three times in IP buffer and the immunoprecipitated complexes were eluted by boiling in sample buffer. Immunoblotting was performed to analyse the precipitated proteins.

### 4.9 RBP IP

Cells were first lysed in nuclear isolation buffer (1.28 M sucrose, 40 mM Tris–HCl pH 7.5, 20 mM MgCl_2_, 4% Triton X‐100, RNAase and protease inhibitors). After that, resuspend nuclear pellet in freshly prepared RBP IP (RIP) buffer (150 mM KCl, 25 mM Tris pH 7.4, 5 mM EDTA, 0.5 mM DTT, 0.5% NP40, RNAase and protease inhibitors) and mechanically shear chromatin using a dounce homogeniser with 15–20 strokes. After centrifugation, the lysis was incubated with anti‐IGF2BP3 or m^6^A antibody or IgG pre‐conjugated protein A/G agarose beads at 4°C overnight. The IP complex was treated with Proteinase K and RNA was purified with phenol:chloroform:isoamyl alcohol. Real‐time PCR was performed and the primers were used as described previously.^11^

### 4.10 RNA dot blot

RNA samples were incubated at 95°C in a heat block to disrupt secondary structures and the tubes were chilled on ice immediately. After denaturation, drop 2 μL RNA onto the positively charged nylon membrane and crosslink RNA to the membrane with UV light: 125 mJoule/cm^2^ at 254 nM. After blocking with non‐fat milk, the membrane was incubated with primary and secondary antibodies and visualised using ChemiDoc MP Imaging System. Methylene blue stain was used to assess RNA immobilised on hybridisation membranes.

### 4.11 Mito‐Keima mitophagy analysis

Cells were transfected with the mKeima‐Red‐Mito‐7 (56018; Addgene) plasmid using Lipo8000™ for 24 h and then treated as indicated. The cells were imaged using Leika TCS SP5 Confocal microscope (Ex = 550 nm, Em = 620 nm for acidic red fluorescence).

### 4.12 Confocal microscope imaging

Cells were first cultured on eight‐well chambered coverglass (C8‐1.5H‐N; Cellvis) overnight, followed by designated treatment. All of the confocal images were obtained with 60× oil objective (numerical aperture 1.4) lenses of Leika TCS SP5 Confocal.

### 4.13 Immunohistochemistry assay

Tumour tissue samples were embedded in paraffin and antigen retrieval was performed. Following the blockade of endogenous peroxidase activity, the samples were incubated with the primary antibodies of interest and the appropriate secondary antibodies and reacted with DAB detection reagents (G1212; Servicebio). H&E staining was used to indicate tumour cells and nuclei of tumour sections.

### 4.14 Mass spectrometry analysis

To identify the ubiquitination site of IGF2BP3, HeLa cells were co‐transfected with GFP‐IGF2BP3, Flag‐Parkin and HA‐ubiquitin, and cell lysates were immunoprecipitated using GFP‐Trap^®^ Agarose. The proteomic sample preparation was performed as described previously.^32^ The peptides were analysed with an LC‐MS/MS system which comprised a Dionex Ultimate 3000 RSLC nano LC system (Thermo Fisher) coupled to a Q‐Exactive mass spectrometer (Thermo Fisher). The UniProt human database was used for data searches using an in‐house Mascot server (version 2.4.1; Matrix Science). Shortlisted ubiquitinated peptides were further confirmed by manual inspection of MS/MS spectra to identify the exact ubiquitination sites. The analysis was performed by Jiyun Biotech.

### 4.15 Reverse transcription and quantitative real‐time PCR

RNA was extracted with the RNeasy kit (217004; Qiagen). A reverse transcription reaction was performed using 1 μg of total RNA with iScript™ Reverse Transcription Supermix for RT‐qPCR (170‐8841; Bio‐Rad). The mRNA expression levels were determined by real‐time PCR using SsoFast EvaGreen Supermix (172‐5201AP; Bio‐Rad) and the CFX96 Touch Real‐time PCR Detection System (Bio‐Rad). GAPDH was used as an internal control of RNA integrity. Real‐time PCR was performed in triplicate.

### 4.16 Transcriptome sequencing

Total RNA was isolated and purified using TRIzol reagent (Invitrogen) following the manufacturer's procedure. The RNA amount and purity of each sample was quantified using NanoDrop ND‐1000 (NanoDrop). The RNA integrity was assessed by Agilent 2100 with RIN number > 7.0. Poly (A) RNA is purified from total RNA (5 μg) using poly‐T oligo‐attached magnetic beads using two rounds of purification. Then the poly (A) RNA was fragmented into small pieces using divalent cations under high temperature. Then the cleaved RNA fragments were reverse‐transcribed to create the cDNA, which were next used to synthesise U‐labelled second‐stranded DNAs with *E. coli* DNA polymerase I, RNase H and dUTP. An A‐base is then added to the blunt ends of each strand, preparing them for ligation to the indexed adapters. Each adapter contains a T‐base overhang for ligating the adapter to the A‐tailed fragmented DNA. Single‐ or dual‐index adapters are ligated to the fragments, and size selection was performed with AMPureXP beads. After the heat‐labile UDG enzyme treatment of the U‐labelled second‐stranded DNAs, the ligated products are amplified with PCR by the following conditions: initial denaturation at 95°C for 3 min; eight cycles of denaturation at 98°C for 15 s, annealing at 60°C for 15 s, and extension at 72°C for 30 s; and then final extension at 72°C for 5 min. The average insert size for the final cDNA library was 300 bp (±50 bp). At last, we performed the 150 bp paired‐end sequencing on an Illumina X Ten (LC Bio, China) following the vendor's recommended protocol.

### 4.17 Colony formation assay

A total of 200 cells were cultured in six‐well plate for 12–20 days in culture medium. Surviving colonies were stained with Gentian Violet after methanol fixation and visible colonies (≥50 cells) were counted. The experiments were performed in triplicate.

### 4.18 Cell proliferation assay

Cells were seeded in a 96‐well plate for 24 h. After that, add 10 μL of CCK‐8 (cell counting kit 8, E606335‐0500; Sangon Biotech) solution to each well of the plate and incubate the plate for 1–4 h in the incubator. Finally, measure the absorbance at 450 nm using a microplate reader.

### 4.19 Tumour spheroids formation

Matrix‐Gel™ (C0382; Beyotime) was used for 3D tumour ball formation experiments. HeLa‐Parkin cells expressing wildtype or mutant IGF2BP3 were first mixed with Matrix‐GEL™ at a ratio of 1:1, respectively, and then added to the 96‐well plate. Until the Gel drops solidified, cell culture medium with 10% FBS was added for 5‐day culture. Tumour spheroidal structures can be formed and detected.

### 4.20 In vivo xenograft tumour model

Four‐week‐old female BALB/c nude mice were purchased from Shanghai SLAC Laboratory Animal Co. Ltd. All experiments were performed in accordance with the official recommendations of Institutional Animal Care and Use Committee (IACUC) in Zhejiang Provincial People's Hospital, and animals received humane care according to the criteria outlined in the ‘Guide for the Care and Use of Laboratory Animals’. A suspension containing 5 × 10^6^ HeLa‐Parkin cells expressing wild‐type or mutant IGF2BP3 were subcutaneously injected into the right flanks of the nude mice. Mice tumour volume was calculated using vernier caliper. Tumour‐bearing mice were killed 2 weeks after inoculation, and xenograft tumours were weighed.

### 4.21 Statistical analysis

All western blotting data and image data presented were representative of three independent experiments. The numeric data were presented as mean ± S.D. from three independent experiments. Student's *t*‐test was used to compare the means of two groups and the ANOVA test was used to compare the means of multiple groups. *p* Value less than 0.05 was considered *p* > 0.05 statistically significant. #*p* > 0.05; *p < 0.05; **p < 0.01; ***p < 0.001 Image J was used for cell fluorescence analysis. At least 10 cells of one image and three images from each group were chosen for Image J analysis.

## CONFLICT OF INTEREST STATEMENT

The authors declare no conflict of interest.

## Supporting information


**Suppl Figure 1**. IGF2BP3 is highly expressed in human cervical cancer and negatively correlates with Parkin expression. A, GEPIA of IGF2BP expression in cancer tissues and noncancer tissues of patients (T = 306 N = 13). B, The correlation of IGF2BP3 expression with the survival of cervical cancer patients. C, The expression levels of Parkin in cancer tissues and noncancer tissues and its correlation with the survival of cervical cancer patients. D, Mass spectrometry analysis showed that IGF2BP1 and IGF2BP2 were listed in the Parkin pulldown complex.
**Suppl Figure S2** IGF2BP1/2 are regulated by the E3 ubiquitin ligase Parkin. A and B, Flag‐Parkin was overexpressed in HeLa cells, and the protein levels of IGF2BP1/2 were analyzed using confocal microscopy. Scale bar 10 μm. C, The fluorescence signals of IGF2BP1 and IGF2BP2 were analyzed using ImageJ, and statistical analysis was performed. D, C33A or MS751 cells were overexpressed with Flag‐Parkin, and cells were lysed for western blotting analysis of IGF2BP1 expression. E, CaSki cells overexpressing Flag‐Parkin or HeLa‐229 cells were transfected with PARK2 siRNA. Western blotting was used to detect the protein levels of IGF2BP2. F, The expression level of IGF2BP3 in Parkin‐overexpressing HeLa cells in the presence of CHX (50 μg/ml) for different times. G, The expression level of IGF2BP3 in mouse hepatocytes overexpressing Parkin in the presence of CHX (50 μg/ml) for different times. H, The protein levels of IGF2BP1/2 were analyzed in Parkin‐overexpressing HeLa cells with or without MG132 (10 μM) treatment.
**Suppl Figure S3** IGF2BP3 is required for Parkin‐mediated mitophagy. HeLa cells were first transfected with IGF2BP3 siRNA and then overexpressed Flag‐Parkin plasmid. The levels of mitochondrial proteins were evaluated by western blotting.
**Suppl Figure S4** In vivo ubiquitination analysis of IGF2BP3 and IGF2BP1. A, HeLa cells were co‐overexpressed with GFP‐IGF2BP3, Flag‐Parkin and HA‐ubiquitin, and cells were lysed for IP. The level of K48‐linkage‐specific polyubiquitin was measured in the pulldown proteins by GFP. B, HeLa cells were co‐overexpressing GFP‐IGF2BP1, Flag‐Parkin and HA‐ubiquitin, and the cells were lysed for IP. The levels of ubiquitin were detected in the pulldown proteins by GFP.
**Suppl Figure S5** Parkin ubiquitination affects its regulation of IGF2BP3. A, Flag‐Parkin was overexpressed in HeLa cells, which were then treated with SAHA (2.5 μM, 12 h). Cells were collected for western blotting analysis of IGF2BP3. B, HeLa cells were first co‐overexpressed with GFP‐IGF2BP3 and Flag‐Parkin and then treated with SAHA (2.5 μM, 12 h). Cells were lysed for IP, and the level of IGF2BP3 was measured in the pulldown proteins by FLAG. C, HeLa cells were co‐overexpressed with GFP‐IGF2BP3 and wild‐type or mutant Parkin, and the cells were collected for RNA‐binding protein IP. PCR was applied to examine the levels of target genes of IGF2BP3. D, As in C, RNA extraction was conducted in cells, and PCR was applied to examine the levels of the indicated genes.
**Suppl Figure S6** MS/MS spectra of the peptide containing ubiquitinated K213. Red color was applied for fragment ions having ubiquitinated lysine.
**Suppl Figure S7** Ubiquitination modification influences the levels of target RNAs of IGF2BP3. A, Confocal imaging of IGF2BP3 in HeLa‐Parkin cells with wild‐type or mutant IGF2BP3 expression. B, The binding of IGF2BP3 to its target RNAs was determined using RNA‐binding protein IP. HeLa‐Parkin cells were overexpressed with wild‐type or mutant IGF2BP3, and PCR was applied to examine the levels of target RNAs in pulldown RNA by IGF2BP3. C, As in B, the levels of target RNAs of IGF2BP3 were evaluated using PCR in HeLa‐Parkin cells transfected with wild‐type or mutant IGF2BP3. D, HeLa‐Parkin cells were first overexpressed with wild‐type or mutant IGF2BP3 and then treated with SAHA (2.5 μM, 12 h). RNA extraction was conducted in cells, and PCR was applied to measure the levels of target RNAs of IGF2BP3. E, As in B, HeLa‐Parkin cells were treated with actinomycin D (5 μg/ml) for different times. The expression levels of the TK1 and FSCN1 genes were examined by PCR. F, Dot blot analysis of the m^6^A RNA level in HeLa‐Parkin cells with wild‐type or mutant IGF2BP3 expression.
**Suppl Figure S8**. The binding of IGF2BP3 to the CRD of the PARK2 gene. A, The consensus GG (m^6^A)C sequence in the 3′ UTR of PARK2 was recognized by IGF2BP3. Four pairs of primers for the PARK2 gene were designed accordingly. B, Sequence information for the wild‐type or mutant CRD of the PARK2 gene.
**Suppl Figure S9**. The proliferative effect of IGF2BP3 is attenuated by mitophagy inhibition. A, SiHa cells were first transfected with GFP‐IGF2BP3 and then transfected with PINK1 shRNA. The cell growth rate was evaluated by CCK‐8. B, Colony formation was applied to examine cell growth as described in A.

**Supplementary Table S1**. LC‐MS/MS analysis of IGF2BP3 ubiquitination sites. The lysine at 213 (K213) of IGF2BP3 was identified as the putative ubiquitination site for Parkin.

## Data Availability

The datasets used and/or analysed during the current study are available from the corresponding author on reasonable request.
