# Supplementary material for: Parkin regulates IGF2BP3 through ubiquitination in the tumourigenesis of cervical cancer
Source: Clin Transl Med. 2023 Oct 25;13(10):e1457. doi: 10.1002/ctm2.1457 (PMC10599278; doi:10.1002/ctm2.1457)
Supplement: Supplementary file 1 — Suppl Figure 1. IGF2BP3 is highly expressed in human cervical cancer and negatively correlates with Parkin expression. A, GEPIA of IGF2BP expression in cancer tissues and noncancer tissues of patients (T = 306 N = 13). B, The correlation of IGF2BP3 expression with the survival of cervical cancer patients. C, The expression levels of Parkin in cancer tissues and noncancer tissues and its correlation with the survival of cervical cancer patients. D, Mass spectrometry analysis showed that IGF2BP1 and IGF2BP2 were listed in the Parkin pulldown complex. Suppl Figure S2 IGF2BP1/2 are regulated by the E3 ubiquitin ligase Parkin. A and B, Flag‐Parkin was overexpressed in HeLa cells, and the protein levels of IGF2BP1/2 were analyzed using confocal microscopy. Scale bar 10 μm. C, The fluorescence signals of IGF2BP1 and IGF2BP2 were analyzed using ImageJ, and statistical analysis was performed. D, C33A or MS751 cells were overexpressed with Flag‐Parkin, and cells were lysed for western blotting analysis of IGF2BP1 expression. E, CaSki cells overexpressing Flag‐Parkin or HeLa‐229 cells were transfected with PARK2 siRNA. Western blotting was used to detect the protein levels of IGF2BP2. F, The expression level of IGF2BP3 in Parkin‐overexpressing HeLa cells in the presence of CHX (50 μg/ml) for different times. G, The expression level of IGF2BP3 in mouse hepatocytes overexpressing Parkin in the presence of CHX (50 μg/ml) for different times. H, The protein levels of IGF2BP1/2 were analyzed in Parkin‐overexpressing HeLa cells with or without MG132 (10 μM) treatment. Suppl Figure S3 IGF2BP3 is required for Parkin‐mediated mitophagy. HeLa cells were first transfected with IGF2BP3 siRNA and then overexpressed Flag‐Parkin plasmid. The levels of mitochondrial proteins were evaluated by western blotting. Suppl Figure S4 In vivo ubiquitination analysis of IGF2BP3 and IGF2BP1. A, HeLa cells were co‐overexpressed with GFP‐IGF2BP3, Flag‐Parkin and HA‐ubiquitin, and cells were lysed for IP. [file CTM2-13-e1457-s001.pptx]

## Slide 1
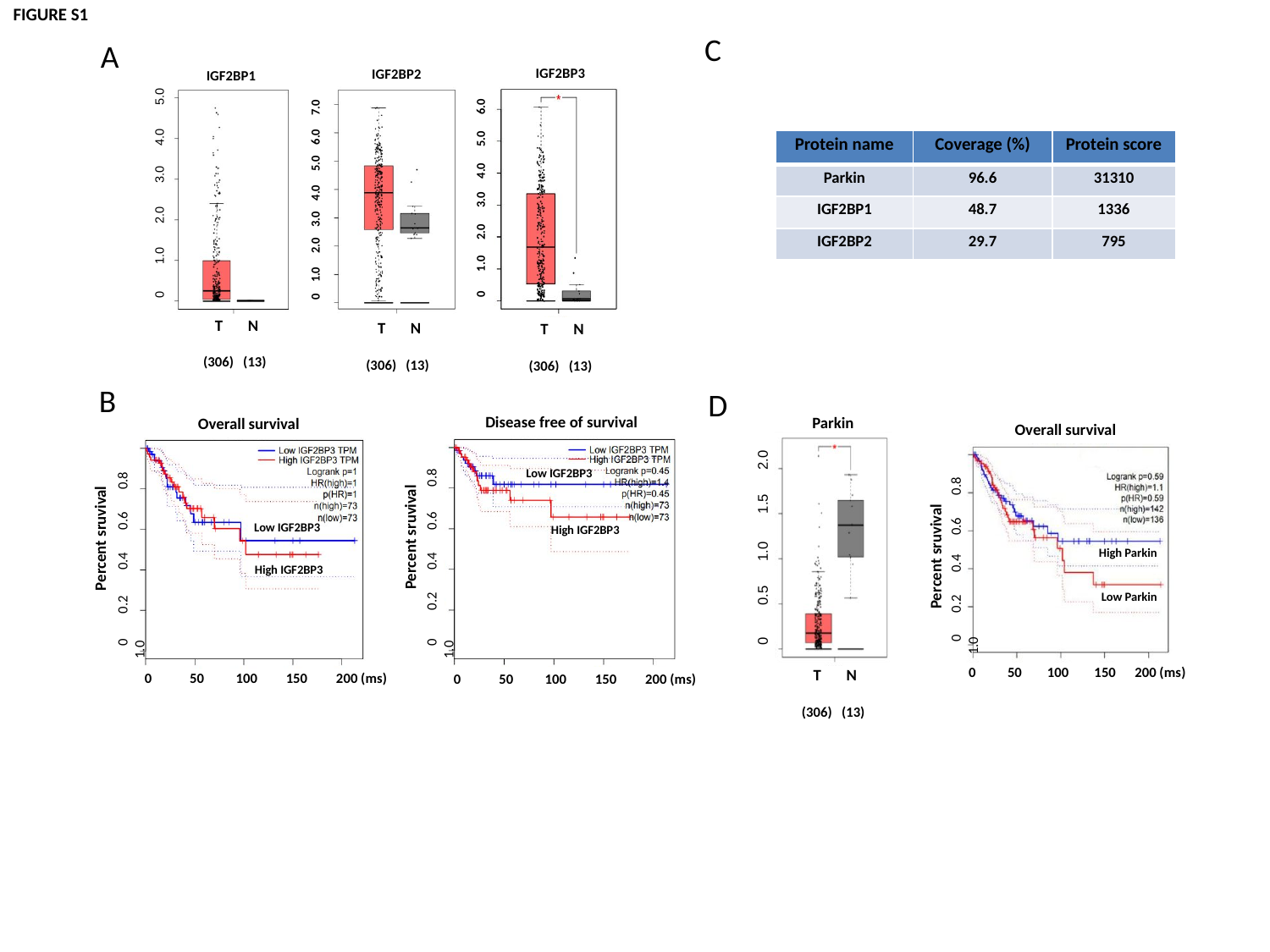

FIGURE S1
C
A
IGF2BP3
0 1.0 2.0 3.0 4.0 5.0 6.0
 T N
 (306) (13)
IGF2BP2
0 1.0 2.0 3.0 4.0 5.0 6.0 7.0
 T N
 (306) (13)
IGF2BP1
0 1.0 2.0 3.0 4.0 5.0
 T N
 (306) (13)
| Protein name | Coverage (%) | Protein score |
| --- | --- | --- |
| Parkin | 96.6 | 31310 |
| IGF2BP1 | 48.7 | 1336 |
| IGF2BP2 | 29.7 | 795 |
B
D
Disease free of survival
 0 0.2 0.4 0.6 0.8 1.0
 Percent sruvival
Low IGF2BP3
High IGF2BP3
 0 50 100 150 200 (ms)
 Overall survival
 0 0.2 0.4 0.6 0.8 1.0
 Percent sruvival
Low IGF2BP3
High IGF2BP3
 0 50 100 150 200 (ms)
Parkin
0 0.5 1.0 1.5 2.0
 T N
 (306) (13)
 Overall survival
 0 0.2 0.4 0.6 0.8 1.0
 Percent sruvival
High Parkin
Low Parkin
 0 50 100 150 200 (ms)

## Slide 2
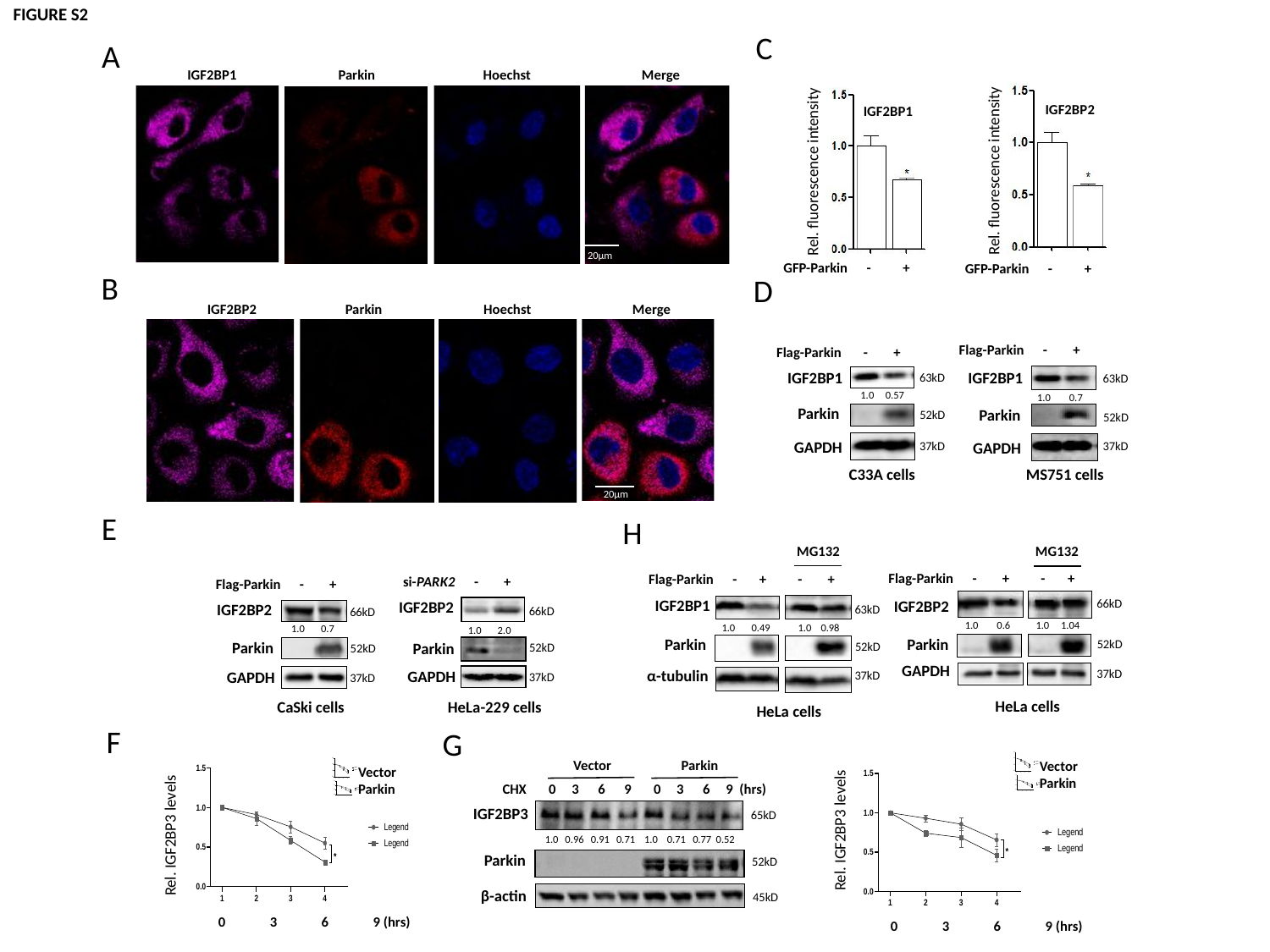

FIGURE S2
C
Rel. fluorescence intensity
GFP-Parkin - +
Rel. fluorescence intensity
GFP-Parkin - +
IGF2BP2
IGF2BP1
A
IGF2BP1 Parkin Hoechst Merge
20μm
B
D
IGF2BP2 Parkin Hoechst Merge
20μm
 Flag-Parkin - +
 Flag-Parkin - +
 IGF2BP1
 IGF2BP1
63kD
63kD
1.0 0.57
1.0 0.7
Parkin
Parkin
52kD
52kD
GAPDH
37kD
GAPDH
37kD
MS751 cells
C33A cells
E
si-PARK2 - +
 Flag-Parkin - +
IGF2BP2
IGF2BP2
66kD
66kD
 1.0 0.7
 1.0 2.0
Parkin
Parkin
52kD
52kD
GAPDH
GAPDH
37kD
37kD
HeLa-229 cells
CaSki cells
H
MG132
MG132
 Flag-Parkin - + - +
 Flag-Parkin - + - +
 IGF2BP1
66kD
IGF2BP2
63kD
 1.0 0.6 1.0 1.04
1.0 0.49 1.0 0.98
Parkin
Parkin
52kD
52kD
GAPDH
37kD
 α-tubulin
37kD
HeLa cells
HeLa cells
F
G
 Vector Parkin
CHX 0 3 6 9 0 3 6 9 (hrs)
IGF2BP3
65kD
1.0 0.96 0.91 0.71 1.0 0.71 0.77 0.52
 Parkin
52kD
45kD
 β-actin
Vector
Parkin
Rel. IGF2BP3 levels
0 3 6 9 (hrs)
Vector
Parkin
Rel. IGF2BP3 levels
0 3 6 9 (hrs)

## Slide 3
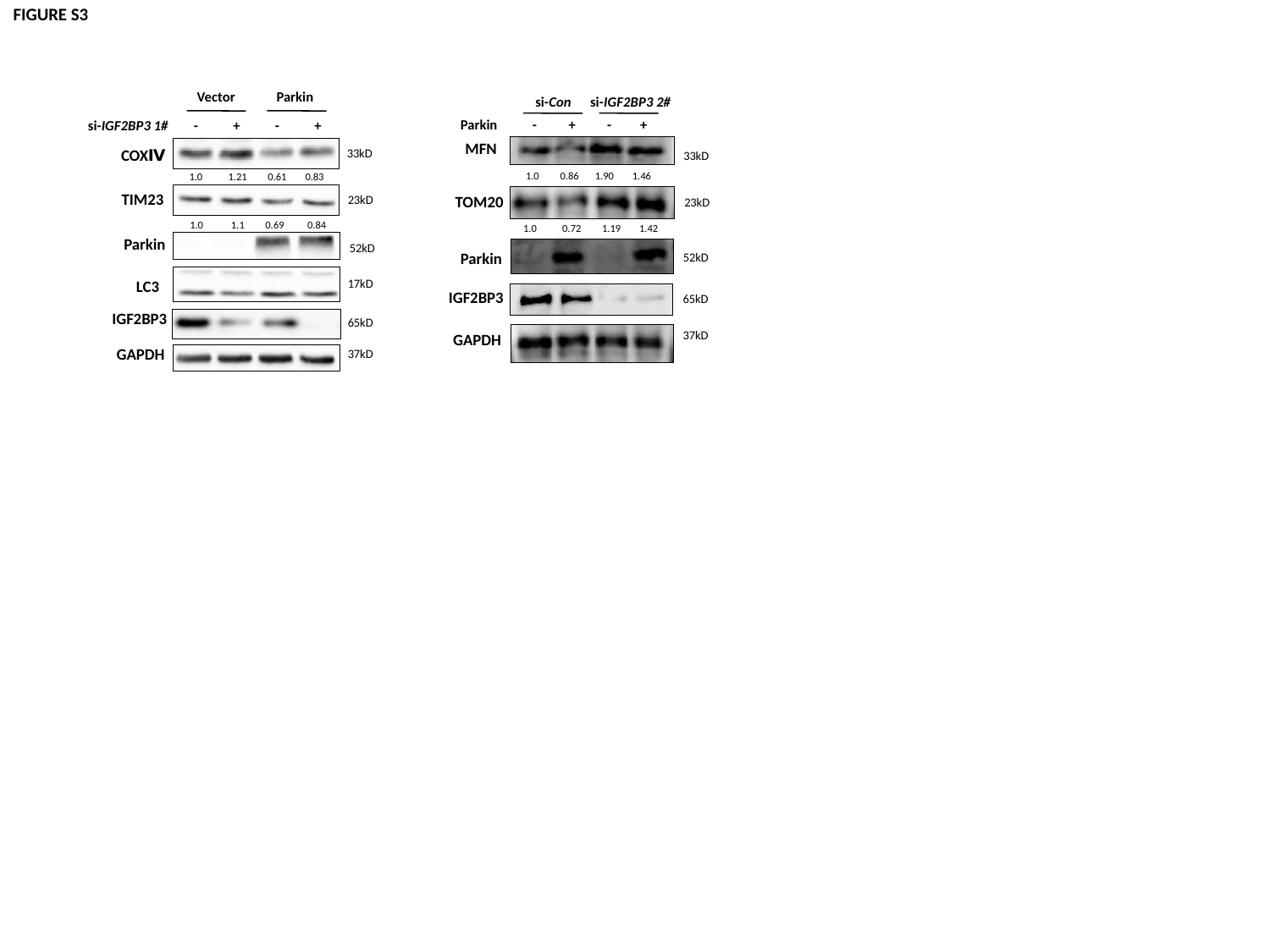

FIGURE S3
Vector Parkin
 si-IGF2BP3 1# - + - +
COXⅣ
33kD
 1.0 1.21 0.61 0.83
TIM23
23kD
1.0 1.1 0.69 0.84
Parkin
52kD
17kD
 LC3
IGF2BP3
65kD
GAPDH
37kD
 si-Con si-IGF2BP3 2#
 Parkin - + - +
MFN
33kD
 1.0 0.86 1.90 1.46
TOM20
23kD
1.0 0.72 1.19 1.42
Parkin
52kD
IGF2BP3
65kD
37kD
GAPDH

## Slide 4
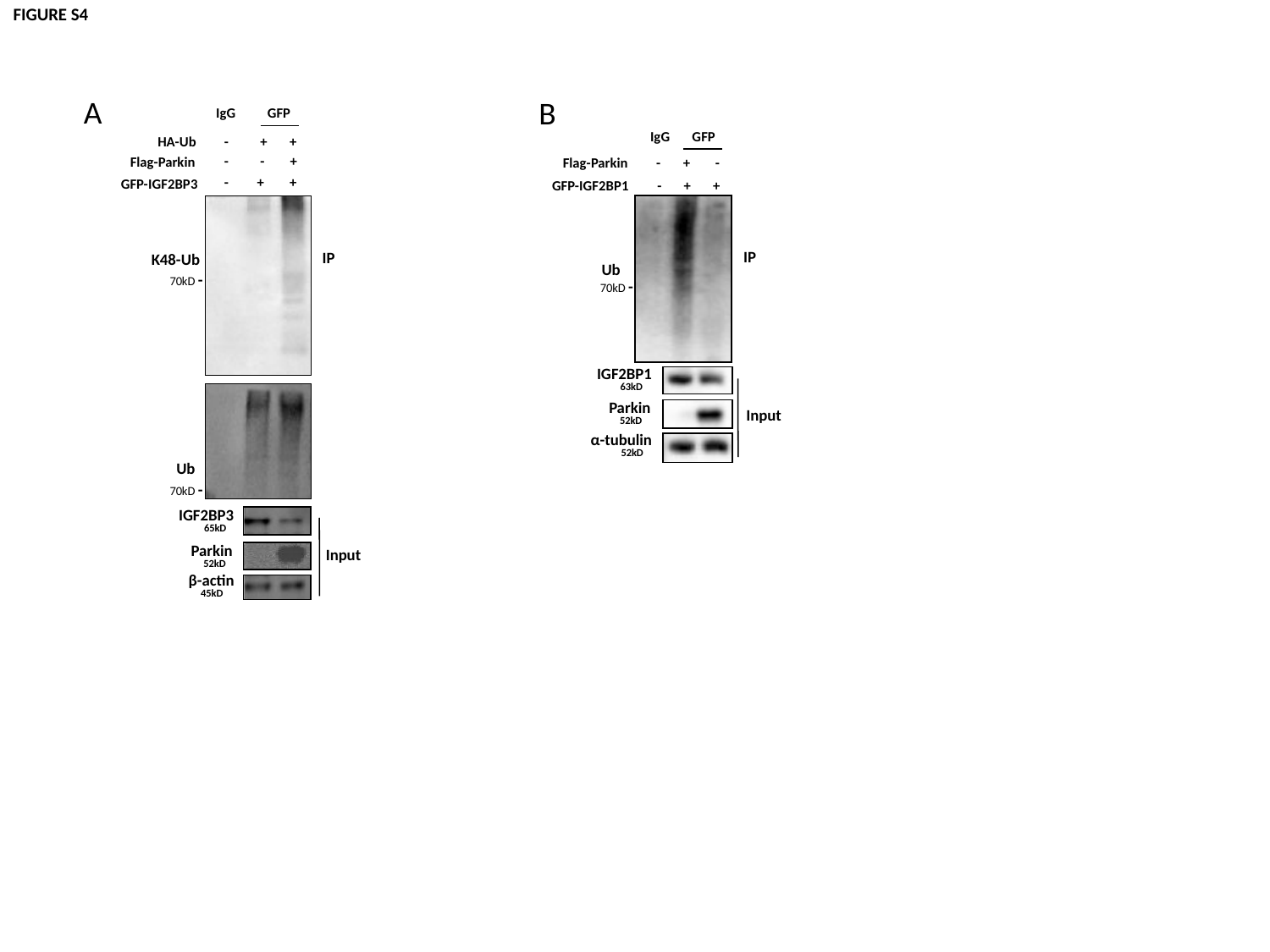

FIGURE S4
A
IgG GFP
 HA-Ub
 - + +
 - - +
Flag-Parkin
 - + +
GFP-IGF2BP3
 IP
 K48-Ub
70kD -
 Ub
70kD -
 IGF2BP3
 65kD
 Parkin
 52kD
Input
 β-actin
 45kD
B
 IgG GFP
Flag-Parkin - + -
GFP-IGF2BP1 - + +
 IP
 Ub
70kD -
 IGF2BP1
 63kD
 Parkin
 52kD
 Input
 α-tubulin
 52kD

## Slide 5
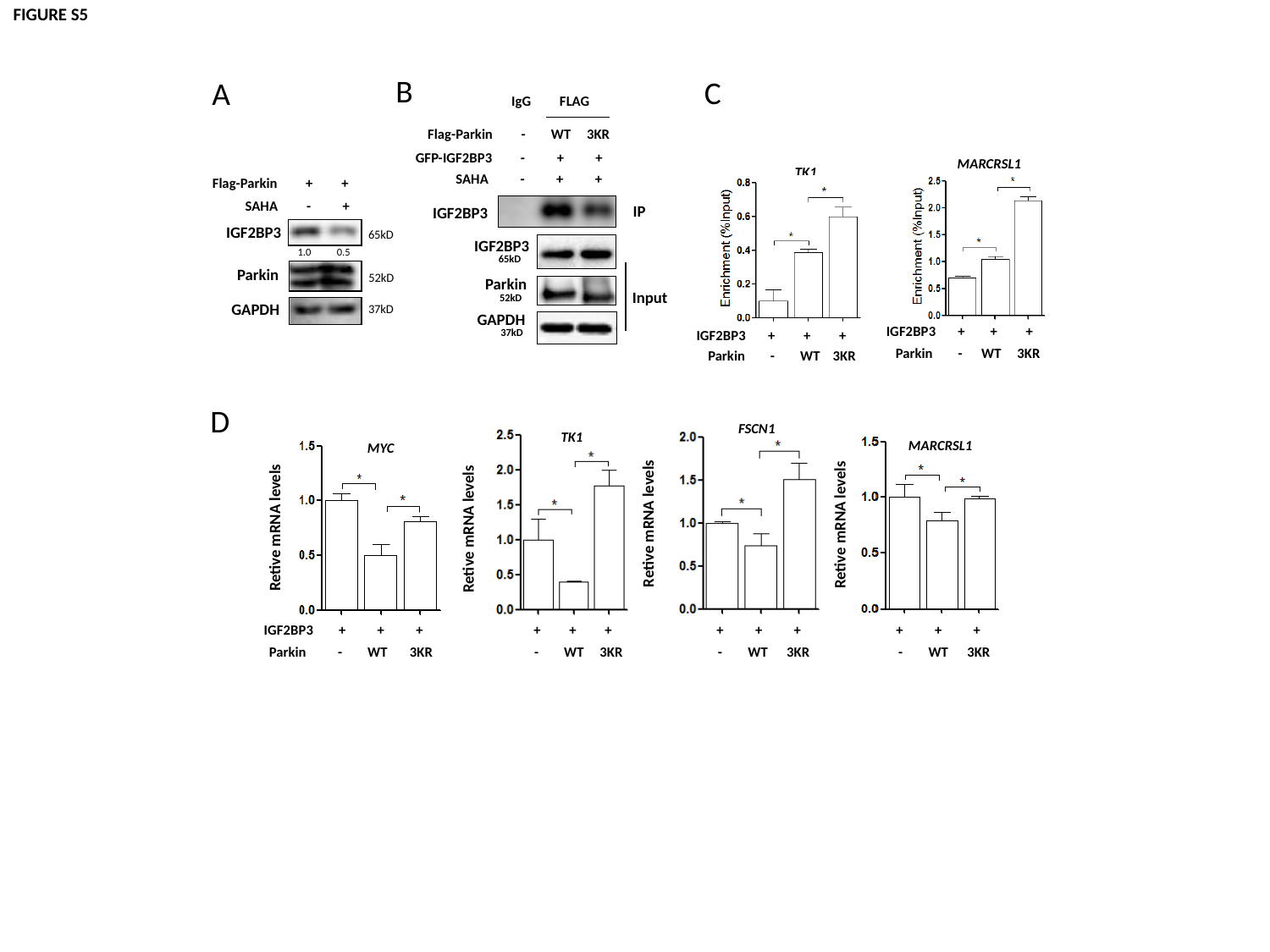

FIGURE S5
B
 IgG FLAG
 Flag-Parkin - WT 3KR
 GFP-IGF2BP3 - + +
 SAHA - + +
 IP
 IGF2BP3
 IGF2BP3
 65kD
 Parkin
 52kD
Input
 GAPDH
 37kD
C
A
MARCRSL1
 IGF2BP3 + + +
 Parkin - WT 3KR
 TK1
 IGF2BP3 + + +
 Parkin - WT 3KR
 Flag-Parkin + +
 SAHA - +
 IGF2BP3
65kD
1.0 0.5
Parkin
52kD
GAPDH
37kD
D
FSCN1
 TK1
Retive mRNA levels
Retive mRNA levels
Retive mRNA levels
MARCRSL1
Retive mRNA levels
MYC
IGF2BP3 + + + + + + + + + + + +
 Parkin - WT 3KR - WT 3KR - WT 3KR - WT 3KR

## Slide 6
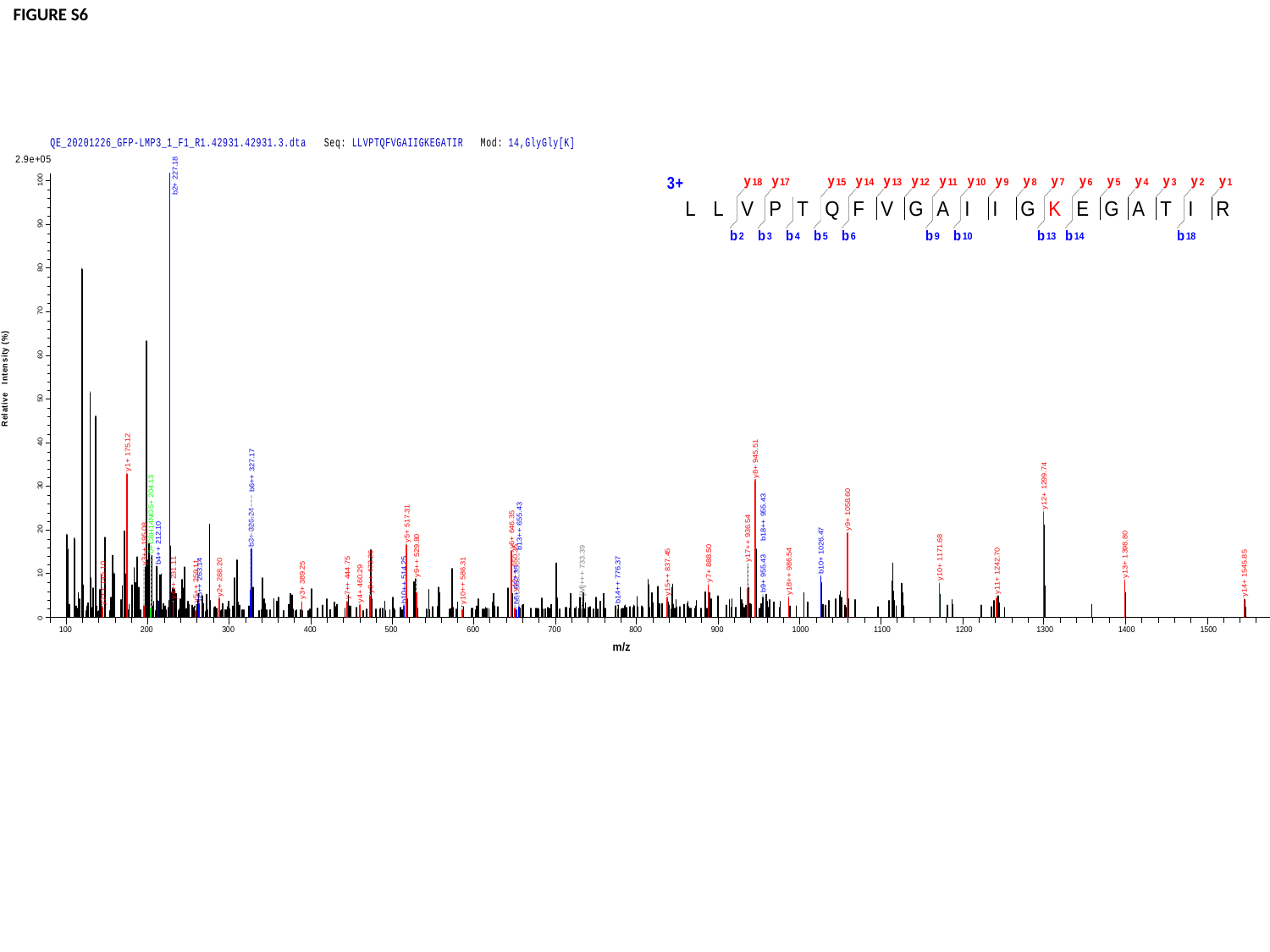

FIGURE S6

## Slide 7
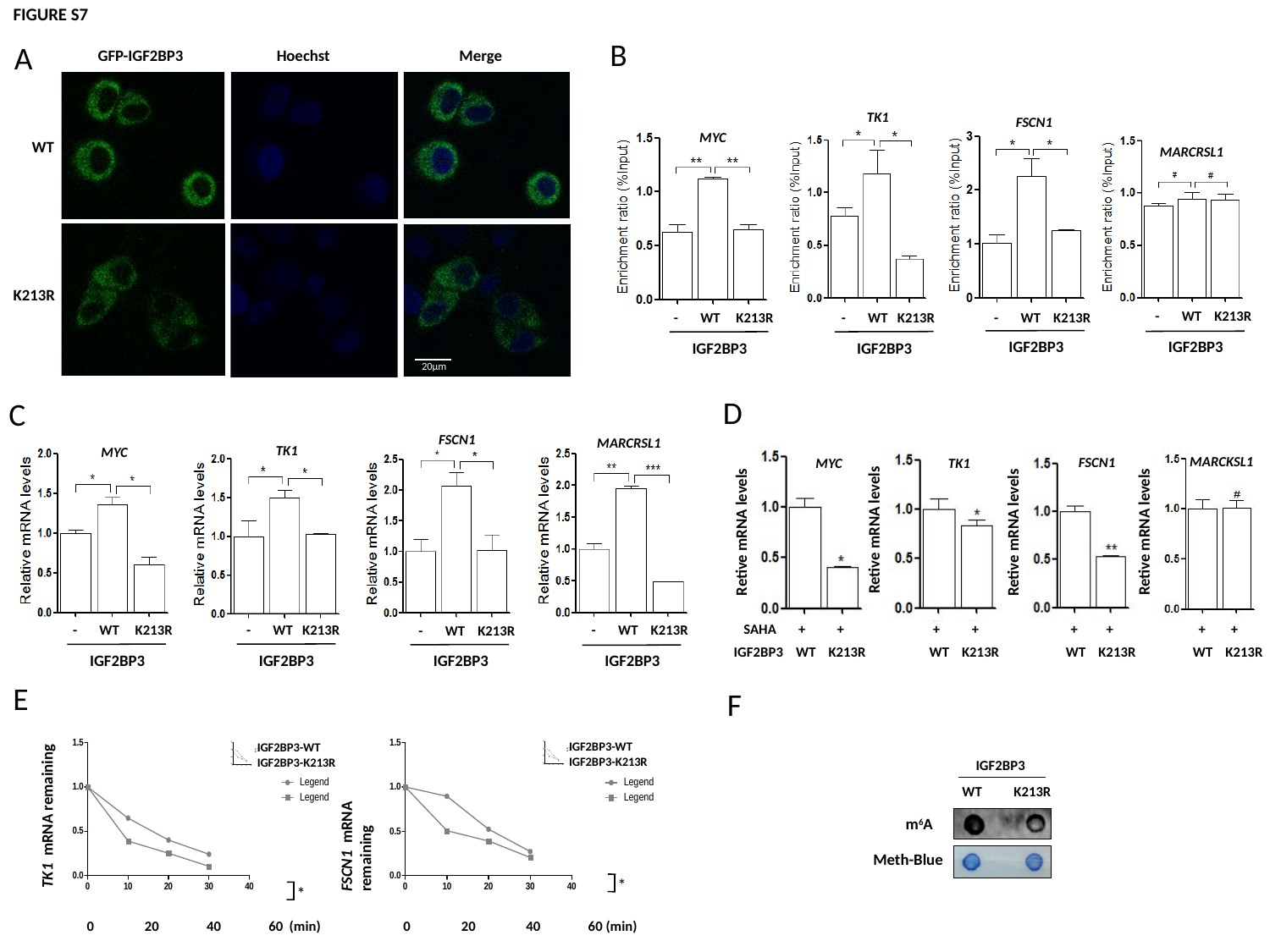

FIGURE S7
B
TK1
FSCN1
MYC
MARCRSL1
 - WT K213R
 - WT K213R
 - WT K213R
 - WT K213R
IGF2BP3
IGF2BP3
IGF2BP3
IGF2BP3
A
 GFP-IGF2BP3 Hoechst Merge
WT
K213R
20μm
D
C
FSCN1
MARCRSL1
TK1
MYC
 - WT K213R
 - WT K213R
 - WT K213R
 - WT K213R
IGF2BP3
IGF2BP3
IGF2BP3
IGF2BP3
Retive mRNA levels
Retive mRNA levels
Retive mRNA levels
Retive mRNA levels
 TK1
MARCKSL1
FSCN1
MYC
SAHA + + + + + + + +
IGF2BP3 WT K213R WT K213R WT K213R WT K213R
E
TK1 mRNA remaining
FSCN1 mRNA remaining
IGF2BP3-WT
IGF2BP3-K213R
IGF2BP3-WT
IGF2BP3-K213R
*
*
0 20 40 60 (min) 0 20 40 60 (min)
F
IGF2BP3
 WT K213R
m6A
Meth-Blue

## Slide 8
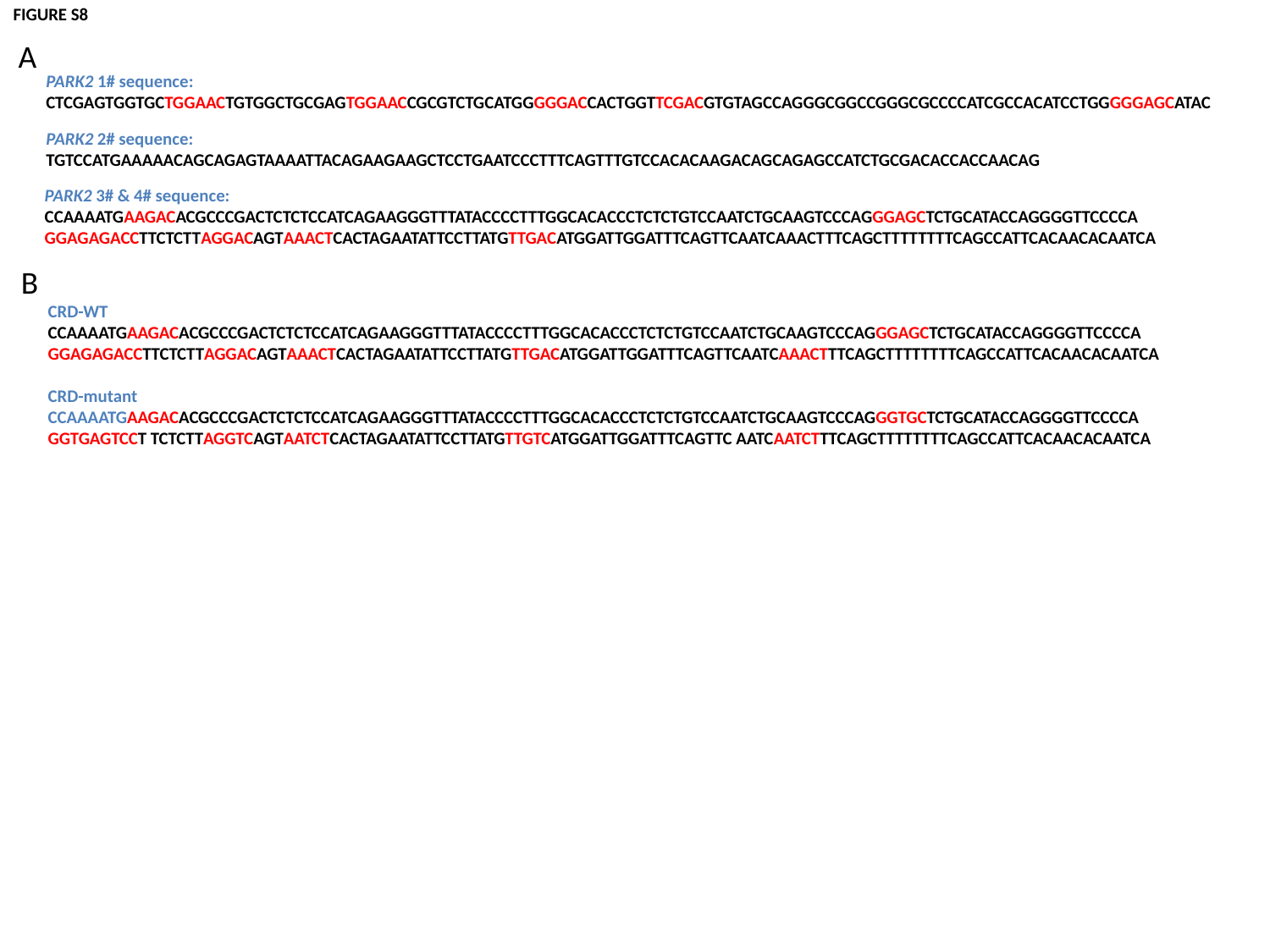

FIGURE S8
A
PARK2 1# sequence: CTCGAGTGGTGCTGGAACTGTGGCTGCGAGTGGAACCGCGTCTGCATGGGGGACCACTGGTTCGACGTGTAGCCAGGGCGGCCGGGCGCCCCATCGCCACATCCTGGGGGAGCATAC
PARK2 2# sequence:
TGTCCATGAAAAACAGCAGAGTAAAATTACAGAAGAAGCTCCTGAATCCCTTTCAGTTTGTCCACACAAGACAGCAGAGCCATCTGCGACACCACCAACAG
PARK2 3# & 4# sequence:
CCAAAATGAAGACACGCCCGACTCTCTCCATCAGAAGGGTTTATACCCCTTTGGCACACCCTCTCTGTCCAATCTGCAAGTCCCAGGGAGCTCTGCATACCAGGGGTTCCCCA
GGAGAGACCTTCTCTTAGGACAGTAAACTCACTAGAATATTCCTTATGTTGACATGGATTGGATTTCAGTTCAATCAAACTTTCAGCTTTTTTTTCAGCCATTCACAACACAATCA
B
CRD-WT
CCAAAATGAAGACACGCCCGACTCTCTCCATCAGAAGGGTTTATACCCCTTTGGCACACCCTCTCTGTCCAATCTGCAAGTCCCAGGGAGCTCTGCATACCAGGGGTTCCCCA GGAGAGACCTTCTCTTAGGACAGTAAACTCACTAGAATATTCCTTATGTTGACATGGATTGGATTTCAGTTCAATCAAACTTTCAGCTTTTTTTTCAGCCATTCACAACACAATCA
CRD-mutant
CCAAAATGAAGACACGCCCGACTCTCTCCATCAGAAGGGTTTATACCCCTTTGGCACACCCTCTCTGTCCAATCTGCAAGTCCCAGGGTGCTCTGCATACCAGGGGTTCCCCA GGTGAGTCCT TCTCTTAGGTCAGTAATCTCACTAGAATATTCCTTATGTTGTCATGGATTGGATTTCAGTTC AATCAATCTTTCAGCTTTTTTTTCAGCCATTCACAACACAATCA

## Slide 9
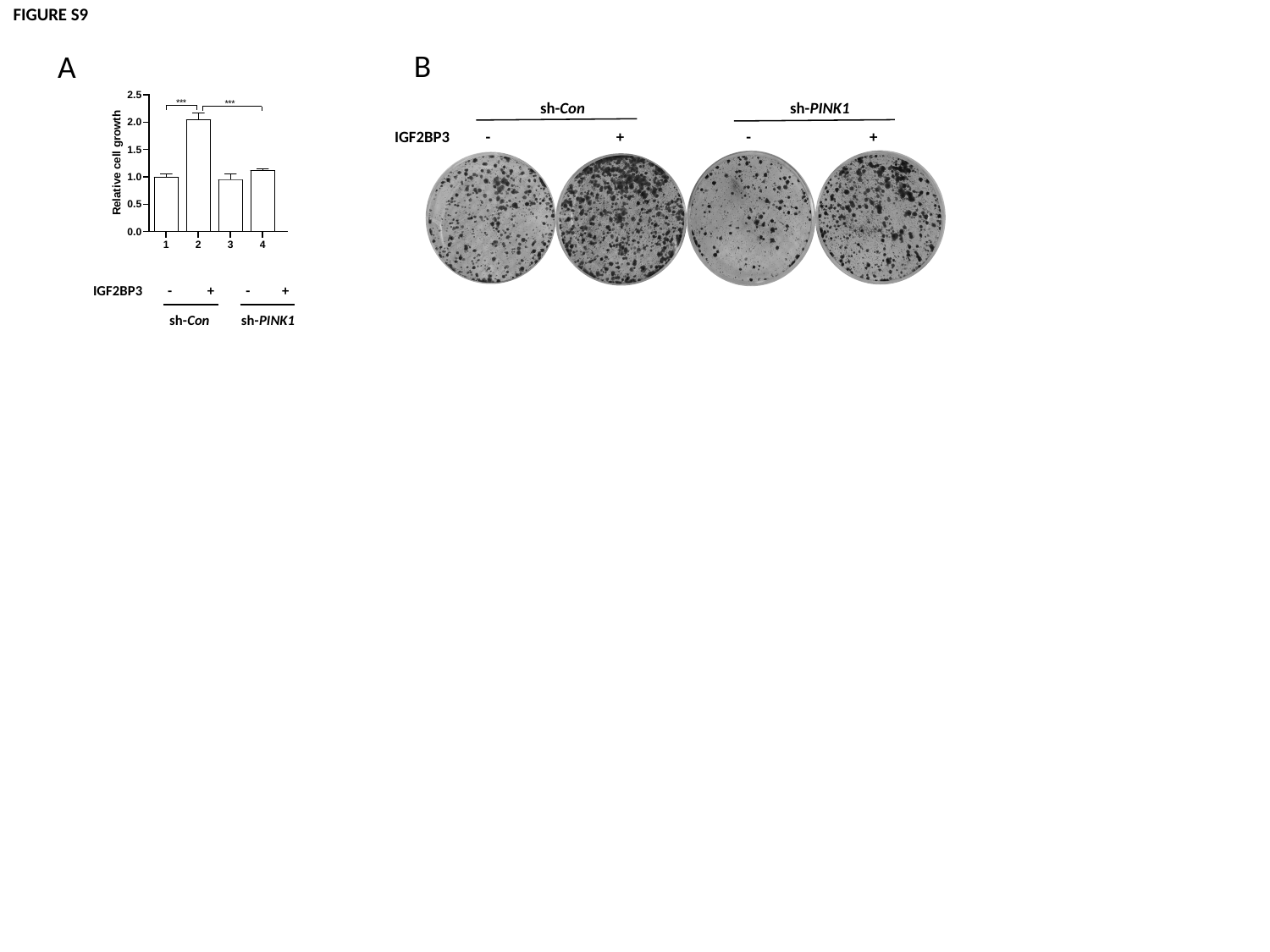

FIGURE S9
B
A
IGF2BP3 - + - +
sh-Con sh-PINK1
 sh-Con sh-PINK1
IGF2BP3 - + - +
